# Mutation of MeCP2 at T158M Leads to Distinct Molecular and Phenotypic Abnormalities in Male and Female Mice

**DOI:** 10.3390/cells14161286

**Published:** 2025-08-19

**Authors:** Chris-Tiann Roberts, Ashraf Kadar Shahib, Khatereh Saei Arezoumand, Ghanan Bin Akhtar, Kazem Nejati-Koshki, Jessica S. Jarmasz, Seyyed Mohyeddin Ziaee, Marjorie Buist, Nicole Raabe, Abbas Rezaeian Mehrabadi, Carl O. Olson, Mojgan Rastegar

**Affiliations:** Biochemistry and Medical Genetics, Rady Faculty of Health Sciences, Max Rady College of Medicine, University of Manitoba, 745 Bannatyne Avenue, Basic Medical Sciences Bldg. Room 627, Winnipeg, MB R3E 0J9, Canada

**Keywords:** MeCP2 mutation, transgenic mice, brain development, DNA methylation, epigenetics, neurodevelopmental disorders, Rett Syndrome

## Abstract

Methyl CpG-binding protein 2 (MeCP2) is an epigenetic reader of DNA methylation with high abundance in the brain. While genetic mutations occur across different protein domains of MeCP2, the T158M mutation is amongst the most frequent MeCP2 mutations. MeCP2 is encoded by the *MECP2*/*Mecp2* gene located on the X chromosome. In humans, *MECP2* mutations cause Rett Syndrome, a debilitating neurodevelopmental disorder in females, with very rare cases presenting in males. Despite the generation of different transgenic mouse lines with MeCP2 mutations, the sex-dependent phenotypic and molecular impact of common MeCP2 mutations in mouse models of disease remains largely unexplored. Here, we focus on the MeCP2 T158M mutation using *Mecp2*^tm4.1Bird/^J transgenic mice (referred to as *Mecp2^T158M^*), and report that *Mecp2^T158M^* mutant mice display sex-specific molecular, behavioural, and phenotypic characteristics when compared to wild-type controls. Our data indicates sex- and brain-region-dependent impacts on the expression of MeCP2, synaptic proteins, cytoskeletal markers, and autophagy factors. Our findings demonstrate that the phenotypic and molecular characteristics of this mouse model may relate to the clinical manifestation in human patients with Rett Syndrome.

## 1. Introduction

Methyl-CpG-binding protein 2 (MeCP2) is an important nuclear protein with key regulatory roles during brain development, from the late embryonic stages into adulthood [1]. An increase in MeCP2 expression during neurodevelopment coincides with key processes in the brain, including neurogenesis, gliogenesis, synaptogenesis, and early circuit formation [1]. During this period, MeCP2 regulates the expression of genes that are involved in neuronal and synaptic maturation. Mutations in the *MECP2* gene account for over 90% of cases of Rett Syndrome (RTT), a debilitating neurodevelopmental and X-linked disorder, which is characterized by a plethora of clinical phenotypes [2]. Eight of the most frequent mutations in *MECP2* comprise both missense and nonsense mutations. The T158M missense mutation of MeCP2 results in disrupted hydrogen bonding and leads to an unstable MeCP2 protein product and may result in globally reduced levels of MeCP2 in the brain [2,3]. MeCP2 deficiency may have a widespread neurological impact that begins early in embryonic development. In human patients, the T158M MeCP2 mutation is associated with altered subcellular localization of nucleolar proteins, suggested to impact ribosome biogenesis, which is important for protein translation [4]. Given the importance of MeCP2 in brain development, altered expression of MeCP2 may influence key neurodevelopmental events. Study of the downstream effects of *MECP2* mutations in the context of RTT may provide important insights into alternative therapeutic avenues.

While RTT mouse models have been extensively studied [5,6], characterization of the molecular and phenotypic properties of relevant mouse models with common MeCP2 mutations has yet to be fully conducted. In addition, an overwhelming number of studies using RTT mice have favoured males, thereby excluding females. RTT patients are primarily females, and affected males may die in utero and rarely survive beyond birth [7]. T158M is one of the most frequent RTT-associated mutations in MeCP2. Therefore, we hypothesize that in a mouse model of this disease, the *Mecp2^T158M^* mutation will lead to similar and specific molecular, phenotypic, and behavioural deficits in RTT mice in a sex-dependent manner. To address our hypothesis, the objective of our study was to assess the behavioural, phenotypic, and brain-region-specific molecular impact of the T158M missense mutation in MeCP2 in male and female *Mecp2*^tm4.1Bird/^J (herein referred to as *Mecp2^T158M^*) RTT mice as compared to their age- and sex-matched wild-type controls. Studies were conducted at the time of the manifestation of RTT symptoms. We first show that the T158M mutation leads to a significant decrease in MeCP2 protein expression in certain brain regions, including the cerebellum, cortex, hippocampus, and thalamus of both male and female RTT mice. Next, we observed that the T158M mutation leads to altered levels of proteins involved in neurodevelopment, such as pre- and post-synaptic proteins and autophagy factors. We further report impaired gait, activity/mobility, general condition/appearance, and breathing, along with other phenotypes, such as hindlimb clasping and tremor, in the mutant mice. Lastly, we studied the behavioural characteristics of the *Mecp2^T158M^* male and female mutant mice compared to age- and sex-matched wild-type control mice by using the elevated plus maze (EPM) and the open field test (OFT). Overall, our results may provide important insights into understanding the molecular, behavioural, and phenotypic effects of MeCP2 mutations in the context of Rett Syndrome and other MeCP2-associated neurodevelopmental disorders.

## 2. Materials and Methods

### 2.1. The Ethical Statement on the Studies

All experimental procedures on live animals in this study (including the protocol for the monitoring and behavioural data, as well as the establishment and maintenance of the transgenic mice) were reviewed and approved by the University of Manitoba Animal Research Ethics Board. All of the reported experimental procedures in mice were completed under our approved animal protocols 18-053 and 23-019. All molecular experiments in this study were reviewed and approved by the Environmental Health and Safety Office (EHSO) at the University of Manitoba.

### 2.2. Mice, Genotyping, and Tissue Collection

The mice were housed in the University of Manitoba Animal Facility with access to food and water ad libitum in an environment that was temperature-controlled at all times. The T158M transgenic mice were purchased from the Jackson Laboratory (B6.129P2(Cg)-*Mecp2*^tm4.1Bird^/J; strain 026762), and the colony was developed and maintained in the University of Manitoba Genetic Modelling Centre (GMC) on a C57BL/6 background. The T158M point mutation was encoded using exon 4 of the *Mecp2* gene, which was tagged with an enhanced green fluorescent protein (EGFP, referred to as GFP hereafter) downstream of the coding sequence in exon 4.

Daily monitoring and phenotypic scoring of the mice were not blinded, as genotyping information was known and associated with the mice’s IDs on data sheets that were used to record relevant information. These data were obtained in collaboration between Central Animal Care Services (CACS) staff (as a paid service) and different lab members for unbiased recording of the data. The Western blot (WB) experiments were not blinded, as the order of the samples in the gels was kept consistent. Phenotypic scoring data for several mice were excluded for the following reasons: (i) mice were found dead before the end of the study or (ii) mice reached the humane endpoint (i.e., 15–20% weight loss). Of note, some mutant mice were found dead *due to* the severity of their symptoms before the end of 20 days of monitoring.

To determine the genotype of each mouse, genomic DNA was isolated from ear punch tissue (samples were collected by the CACS and transferred to our lab for genotyping) using the Invitrogen Pure link Genomic DNA Mini Kit DNA extraction kit, as instructed by the supplier (Invitrogen, Carlsbad, CA, USA). An experimental polymerase chain reaction (PCR) was completed by using a common forward primer [5′-CAG CAG CAT CTG CAA AGA AG-3′] for both the wild-type and mutant allele, but two separate reverse primers were used: one specific to the wild-type allele [5′-CTG GTC AAC AGC TTG TCT GG-3′] and the other to the mutant allele [5′-CTG AAC TTG TGG CCG TTT AC-3′] [8]. PCR reactions were prepared in a 15 µL reaction volume containing genomic DNA, primers, Master Mix (Platinum II Green HS PCR MM (2X)), and high pure commercial H_2_O. The PCR products were separated on a 1.5% agarose gel containing SYBR Safe DNA Gel Stain (Life Technologies Inc., Carlsbad, CA, USA) and visualized under UV light. Genotypes were determined based on the presence or absence of bands corresponding to the wild-type or mutant allele. Samples with bands from both primer sets were identified as heterozygous.

Experiments with the wild-type and hemizygous male *Mecp2^T158M^* were completed at 5–9 weeks of age (depending on the type of test), and experiments with the wild-type and heterozygous females *Mecp2^T158M^* were completed at 15–19 weeks of age (depending on the type of test). For all studies, age- and sex-matched controls were used. Each mouse was weighed and scored for its RTT-like phenotype (Supplementary Table S1) daily for 20 days and then euthanized via a CO_2_ overdose. The Supplementary Table S1 was prepared based on other reported in vivo studies [9,10,11], with modifications. The brains were weighed and their lengths measured (from the anterior olfactory bulbs to the posterior cerebellum) before dissection. One brain hemisphere was dissected for protein extraction, snap-frozen, and stored in a −80 °C freezer. The other hemisphere was fixed for 20 min in freshly made ice-cold de-polymerized paraformaldehyde (2% PFA in 0.16 M sodium phosphate buffer, pH = 7.4); rinsed twice with cold 0.05 M phosphate-buffered saline (PBS); and then stored in cryoprotectant (25 mM sodium phosphate buffer, pH = 7.4, 10% sucrose, 0.02% sodium azide) at 4 °C, as previously reported [12,13]. After PFA fixation, the tissue samples were embedded into the Tissue-Tek^®^ OCT compound (Electron Microscopy Sciences, Hatfield, PA, USA) to facilitate cryopreservation. The brain tissues were then cryosectioned at a thickness of 14–16 μm using a precision cryostat (Leica Biosystems, Vista, CA, USA). The resulting sections were immediately stored at −80 °C to preserve tissue integrity for downstream analyses.

### 2.3. Protein Extraction and Western Blots

Protein extraction from specific brain regions and quantification of the extracted proteins were completed as previously described [14]. Protein extracts from the collected brain tissues (25 µg) were subjected to Western blotting using specific primary and secondary antibodies (Supplementary Table S2), as we previously reported [14,15].

### 2.4. RNA Extraction, DNase I Treatment, cDNA Synthesis, and Real-Time RT-PCR Analysis

Total RNA was extracted from specific brain regions using the Trizole extraction method according to the manufacturer’s instructions (Life Technologies Inc., Carlsbad, CA, USA) [16]. Total RNA was quantified (NanoDrop 2000c; ThermoFisher Scientific, San Jose, CA, USA) and subjected to DNase I treatment to eliminate any genomic DNA contamination using the Ambion TURBO DNA-free kit (ThermoFisher Scientific, San Jose, CA, USA). cDNA synthesis was performed using a Superscript II Reverse Transcriptase kit (Invitrogen, CA, USA) according to the supplied instructions. For quantitative RT-PCR, a total volume of 20 µL was used, including the cDNA, primers (forward and reverse), DNase/RNase-free water, and *Power*SYBR Green-based RT-PCR Master Mix reagent (Applied Biosystems, Foster City, CA, USA). All samples were run in technical duplicate with three different biological replicates [17]. The qPCR reactions were carried out on a 7500 Fast Real-Time PCR machine (Applied Biosystems, Foster City, CA, USA). The transcript levels of the *Mecp2* isoforms (e1 and e2), *Bdnf* (brain-derived neurotrophic factor), and *Gapdh* (glyceraldehyde 3-phosphate dehydrogenase) were analyzed. The sequence of the primers used in the qRT-PCR reactions was as follows: *Mecp2e1*: forward: 5′-AGG AGA GAC TGG AGG AAA AGT-3′, reverse: 5′-CTT AAA CTT CAG TGG CTT GTC TCT G-3′ [18]; *Mecp2e2*: forward: 5′-CTC ACC AGT TCC TGC TTTGAT GT-3′, reverse: 5′-CTT AAA CTT CAG TGG CTT GTC TCT G-3′ [18]; *Bdnf*: forward: 5′-GCG CCC ATG AAA GAA GTA AA-3′, reverse: 5′-TTC GAT GAC GTG CTC AAA AG-3′ [19]; and *Gapdh*: forward: 5′-AAC GAC CCC TTC ATT GAC-3′, reverse: 5′-TCC ACG ACA TAC TCA GCA C-3′ [18].

### 2.5. Immunohistochemistry (IHC)

Immunohistochemistry with fluorescence staining was carried out as described previously (with minor modifications) [12]. Briefly, slides containing three equally spaced 14–16 µm brain sections at room temperature (RT) (approximately 22 °C) were dried for 30 min prior to 1X PBS incubation for 5 min. To re-permeabilize the tissue, the slides were incubated with 1% Triton-X-100 in 1X PBS for 5 min, followed by two more washes with 1X PBS (5 min). Non-specific binding was blocked by adding blocking solution (5% milk, 1% BSA, + 0.3% Triton-X in PBS) for one hour. After blocking, the slides were incubated with the primary antibody (prepared in the same blocking solution) overnight at 4 °C, followed by incubation with the secondary fluorescent antibody for one hour (RT) (Supplementary Table S2). After incubation with each antibody, four washes with 1X PBS (10 min) were performed. The slides were then incubated with DAPI (1:10,000 prepared in 1X PBS) for 30 min at room temperature and subjected to two 1X PBS washes (10 min); received ProLong Antifade Mountant (Life Technologies, Carlsbad, CA, USA); and were coverslipped (Brand Deckglaser micro 24 × 60 mm, Fisher Scientific, San Jose, CA, USA) and left to dry overnight. Single tile or Z-stacked images were captured using a Zeiss Observer Z.1 inverted microscope (Zeiss, Oberkochen, Germany) with the Zen 3.03 software at 20×, 40×, or 63× magnification. To validate the antibody specificity, both negative and positive controls were employed. In the negative controls, the primary antibody was absent from the slides during the staining protocol (primary omission), while for the positive controls, tissues known to express the target antigen were utilized. All of the samples were processed simultaneously under identical experimental and imaging conditions to ensure consistency. Wild-type samples were used to determine the optimal exposure times before all slides were imaged. Images were subjected to brightness and contrast changes to adjust the background. These changes were applied equally to all images.

### 2.6. Behavioural Testing

During the final days of monitoring (days 17–20), the mice were subjected to behavioural testing in a darkened tent. The testing apparatus was placed in the middle of the tent and illuminated with four infrared lights and a camera positioned directly above it. All mice were relocated from their home cages to the behaviour testing room. Each mouse was tracked (centre-point, nose-point, and tail-base detection) and video-recorded using the EthoVision XT software (Version 16.0.1538, July 2021 by Noldus Information Technology, Wageningen, The Netherlands). After the behavioural recording, each mouse was returned to its home cage, and the testing apparatus was wiped clean with 10% ethanol in preparation for the next mouse. All mice were given 24 h of resting time before the next behaviour test.

#### 2.6.1. The Elevated Plus Maze

The elevated plus maze (EPM; Panlab Harvard Apparatus, CA, USA) evaluates anxiety-like behaviour and exploratory drive in rodents [20,21]. The plus sign (+)-shaped apparatus consists of two open (65 cm total) and two closed arms of an equal size (65 cm total length; 15 cm height of the wall) connecting to a central platform (6 cm × 6 cm) that is 40 cm above the ground [20]. When starting the test, the mouse was placed on the centre part facing a closed arm and was tracked and video-recorded for 10 min (600 s). The data collected included time and frequency in open arms 1 and 2, and closed arms 1 and 2, along with distance travelled (cm) and velocity (speed; cm/s). Tracking and heat maps were also produced. After 10 min of recording, the mouse was returned to its home cage, and the apparatus was wiped clean with 10% ethanol in preparation for the next subject. For the elevated plus maze, the mean percentage of time spent in the open arms (open arm 1 + open arm 2/total time all four arms) × 100) as well as in the closed arms (closed arm 1 + closed arm 2/total time all four arms) × 100) was calculated.

#### 2.6.2. The Open Field Test

The open field test (OFT) has been used to study motor function (locomotion) as well as exploratory drive in rodents [21,22]. The apparatus consists of a 60 cm × 60 cm × 60 cm (W × D × H) white opaque box which rests on a white platform directly on the floor. At the start of the test, the mouse was placed facing the wall and tracked and video-recorded for 10 min (600 s). The data collected included time and frequency in the centre as well as corners 1, 2, 3, and 4 along with distance travelled (cm) and velocity (speed: cm/s). Tracking and heat maps were also produced. After 10 min of recording, the mouse was returned to its home cage, and the apparatus was wiped clean with 10% ethanol in preparation for the next subject. For the open field, the mean percentage of time spent in the centre (centre/total time) × 100) as well as in the four corners (corner 1 + corner 2 + corner 3 + corner 4/total time) × 100) was calculated. In this case, the total time represented the sum of the centre and all four corners.

### 2.7. Experimental Groups for Specific Experiments and Statistics

For the measurements of the weights and lengths of the dissected mouse brains, the statistical differences between the means of different categories of measurements were calculated using unpaired *t*-tests; graphed using GraphPad Prism Software; and reported as the mean ± SEM. For mouse brain weight, the experimental groups included male wild-type (*n* = 7), male hemizygous *Mecp2^T158M^* (*n* = 11), female wild-type (*n* = 12), and female heterozygous *Mecp2^T158M^* (*n* = 7) mice. For mouse brain length, the experimental groups included male wild-type (*n* = 7), male hemizygous *Mecp2^T158M^* (*n* = 10), female wild-type (*n* = 12), and female heterozygous *Mecp2^T158M^* (*n* = 7) mice.

WB signals were analyzed and quantified by using Image J software (version 1.53). Statistical analyses of the WB signals were carried out using GraphPad Prism Software and reported as the mean ± standard error of the mean (SEM) [23]. For the WB studies, the experimental groups include male wild-type (*n* = 3), male hemizygous *Mecp2^T158M^* (*n* = 3), female wild-type (*n* = 3), and female heterozygous *Mecp2^T158M^* (*n* = 3) mice.

The RNA transcript levels were calculated using the delta Ct (∆Ct) values for each primer set normalized to *Gapdh*, followed by the ∆∆Ct method in order to calculate the fold change. Statistical analyses were carried out using GraphPad Prism Software and the results reported as the mean ± SEM, where unpaired *t*-tests were used for each brain region. The experimental groups included male wild-type (*n* = 3) and male hemizygous *Mecp2^T158M^* (*n* = 3) mice.

For each IHC experiment, three coronal brain sections were quantified per animal. These sections were spaced at 100 μm intervals to ensure a consistent anatomical representation across samples. IHC intensity measurements (normalized to the area) were made using Image J software (version 1.53). The values are reported as the mean ± SEM. The reported statistical differences between the wild-type (*n* = 4–5) and *Mecp2^T158M^* (*n* = 4–5) mice were calculated using unpaired *t*-tests and graphed using GraphPad Prism Software.

Data from the behavioural tests was subjected to a statistical analysis using GraphPad Prism Software. A statistical analysis via a two-way ANOVA was done with Tukey’s multiple comparisons post hoc analysis test. All of the data shown is the mean ± SEM. For the EPM, the experimental groups included male wild-type (*n* = 12), male hemizygous *Mecp2^T158M^* (*n* = 15), female wild-type (*n* = 17), and female heterozygous *Mecp2^T158M^* (*n* = 10) mice. For the OFT, the experimental groups included the male wild-type (*n* = 10), male hemizygous *Mecp2^T158M^* (*n* = 13), female wild-type (*n* = 13), and female heterozygous *Mecp2^T158M^* (*n* = 6) mice (unless otherwise stated in the figure legend).

Data from the phenotypic scoring tests was subjected to a statistical analysis utilizing GraphPad Prism Software. For the daily and overall phenotypic criteria, an unpaired *t*-test was conducted. The data shown is the mean ± SEM. The experimental groups included male wild-type (*n* = 10), male hemizygous *Mecp2^T158M^* (*n* = 9), female wild-type (*n* = 10), and female heterozygous *Mecp2^T158M^* (*n* = 8) mice.

To improve the interpretation of the results, outliers were identified and subsequently removed prior to the final statistical analysis. Outlier identification was applied to brain weight and length data, as well as behavioural data. Outliers were identified as any value that was more than ± 1.5 standard deviations different from the mean [24,25]. The final numbers for each experimental group (after outlier removal) are reported here.

All statistical analyses that are presented here were performed by using GraphPad Prism software (Version 7 or 10).

## 3. Results

### 3.1. Gross Brain Features of the Mecp2^T158M^ Male and Female Mice

RTT is a severe neurodevelopmental disorder with molecular, phenotypic, and neuropathological effects. Post-mortem analyses of brains from human RTT patients indicate an estimated 12–34% reduction in the brain’s weight, as well as a reduced brain size and volume [26,27]. Compared to the brains from human RTT patients, the reduced regional volumes of the cingulate, frontal, motor and sensory cortices and the smaller striatum, thalamus, and white matter tracts observed in patients are recapitulated in *Mecp2*-deficient mice [28]. Here, we measured the average brain weight and length in the wild-type and *Mecp2^T158M^* mice. We chose male mice at 7–9 weeks of age (RTT-like symptoms begin at 5–6 weeks of age) and female mice at 18–19 weeks of age (RTT-like symptoms begin at 16–17 weeks of age). As expected, the hemizygous *Mecp2^T158M^* male and heterozygous female RTT mice had statistically significantly lower brain weights compared to those in the wild-type control mice (Supplementary Figure S1). The mean brain weight of the male *Mecp2^T158M^* mice (402.0 mg) was 13% lower than that in the wild-type males, and the mean brain weight of the female *Mecp2^T158M^* mice (434.2 mg) was 8% lower compared to that in the wild-type females. Moreover, the brain lengths in the male and female *Mecp2^T158M^* mice were significantly less than those in the wild-type males (**** *p* < 0.0001) and wild-type females (* *p* < 0.05), respectively (Supplementary Figure S1). Collectively, these results suggest that the *Mecp2^T158M^* mouse model may recapitulate the gross neuropathological findings observed in RTT patients and *Mecp2*-deficient mice.

### 3.2. Investigating the Sex-Dependent Protein Expression Profiles in the Brain in Male and Female Mecp2^T158M^ Mice

MeCP2 is detected in different regions of human and murine brains [13,29]. Generally, MeCP2 levels increase postnatally, despite its enriched neuronal expression compared to that in other brain cell types [12,18,30,31]. It has been reported that the GFP-tagged MeCP2 expression in the brains of T158M male mice is about 30% of the GFP-tagged wild-type MeCP2 [32]. It is also well documented that the MeCP2 T158M mutation results in decreased protein stability in vivo in mice [3]. Given that MeCP2 is an important epigenetic regulator in the brain, an improper dosage of this protein may promote the aberrant expression of other genes. Thus, we measured the MeCP2 expression in specific brain regions.

**Figure 1:**
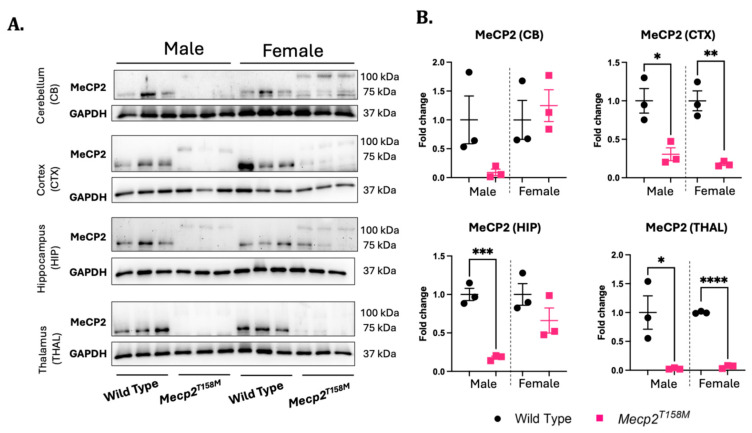
MeCP2 protein levels are reduced in specific brain regions of hemizygous male and heterozygous female *Mecp2^T158M^* mice compared to those in wild-type control mice. (**A**) The detection of MeCP2 by Western blot (with an MeCP2 antibody against the C-terminal of MeCP2) in the cerebellum (CB), cortex (CTX), hippocampus (HIP), and thalamus (THAL) in hemizygous male and heterozygous female *Mecp2^T158M^* mice compared to that in wild-type controls. (**B**) Quantification of detected signals was completed through normalization of the relative signals of the corresponding GAPDH. Fold change values were calculated relative to the average wild-type fold change in the corresponding sex. Bands at 75 kDa correspond to wild-type MeCP2, while bands at 100 kDa correspond to mutated MeCP2. The statistical analysis was performed using an unpaired *t*-test and the results presented as the mean ± SEM (*n* = 3). The levels of significance are shown as * *p* < 0.05, ** *p* < 0.01, *** *p* < 0.001, **** *p* < 0.0001. Uncropped images are shown in Supplementary Figure S2.

In line with previous studies, MeCP2 was significantly reduced in the cortex (* *p* < 0.05), hippocampus (*** *p* < 0.001), and thalamus (* *p* < 0.05) of the hemizygous *Mecp2^T158M^* male mice, about three-, four-, and six-fold, respectively (Figure 1). Similarly, the MeCP2 protein levels were significantly reduced in the cortex (** *p* < 0.01) and thalamus (**** *p* < 0.0001) of the heterozygous *Mecp2^T158M^* female mice compared to those in the controls (Figure 1). A trend of reduction was also observed in the hippocampus of the *Mecp2^T158M^* female mice compared to this value in the controls that was insignificant (Figure 1). In the cerebellum, no significant change was found, although MeCP2 levels showed a trend towards a reduction in the hemizygous male *Mecp2^T158M^* mice and a trend of a slight increase in the heterozygous female *Mecp2^T158M^* mice (Figure 1). These results suggest that the male and female RTT mice used in this study have reduced MeCP2 protein levels in different parts of the brain.

**Figure 2:**
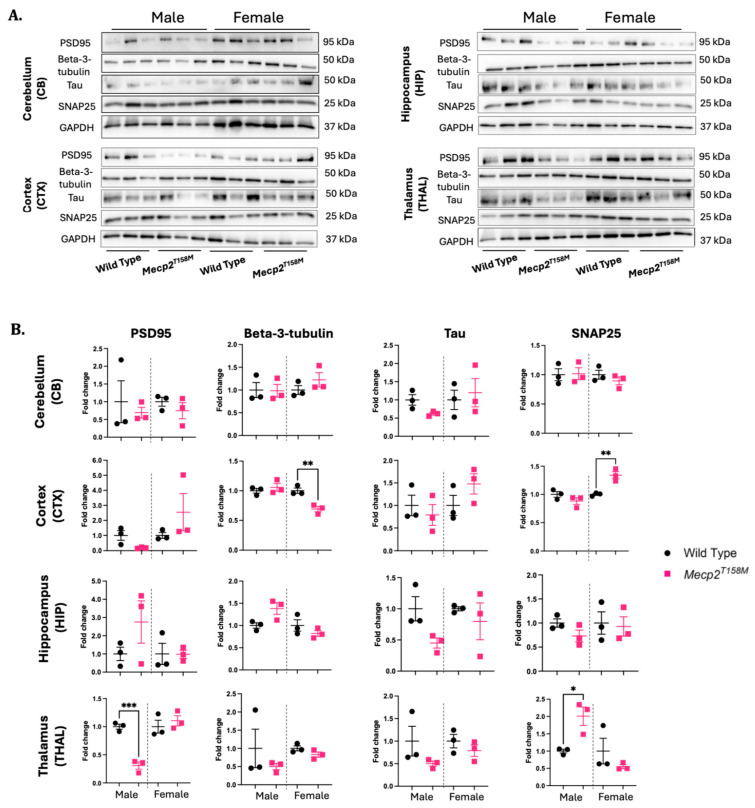
The expression levels of indicated proteins in specific brain regions of the hemizygous male and heterozygous female *Mecp2^T158M^* mice and wild-type control mice. (**A**) Detection of PSD95, beta-3 tubulin, Tau, and SNAP25 by Western blot in the cerebellum (CB), cortex (CTX), hippocampus (HIP), and thalamus (THAL) of male and female *Mecp2^T158M^* and wild-type control mice. (**B**) Quantification of WB signals was carried out by normalizing them to the relative GAPDH signals. Fold changes were calculated relative to the average wild-type fold change in the corresponding sex. Representative GAPDH is shown for the set of Western blots for each brain region. The statistical analysis was performed using an unpaired *t*-test and the results presented as the mean ± SEM (*n* = 3). The level of significance is shown as * *p* < 0.05, ** *p* < 0.01, *** *p* < 0.001. Uncropped images are shown in Supplementary Figure S3.

Human brain cells in RTT are characterized by alterations in the structural properties of the neurons and decreased dendritic complexity, as well as immature synaptic spine morphology [33,34]. Interestingly, diminished connectivity between the cortical excitatory glutamatergic pyramidal neurons is observed in RTT mouse models [35,36]. Considering that MeCP2 plays important roles in synapse formation, these neuropathological features in the presence of MeCP2 mutations may hint to disrupted expression of the proteins involved in synaptic plasticity [37]. In *Mecp2*-deficient mice, the expression of PSD95, a post-synaptic scaffolding protein of the excitatory neurons, was significantly reduced in the hippocampal neurons [37]. In our present study, however, the PSD95 expression was not significantly affected in the hippocampus of the hemizygous male or heterozygous female *Mecp2^T158M^* mice when compared to that in the wild-type controls. However, PSD95 was significantly reduced (*** *p* < 0.001) in the thalamus of the hemizygous male *Mecp2^T158M^* mice compared to this value in the wild-type controls. A similar change was not detected in the heterozygous female *Mecp2^T158M^* mice or the other brain regions tested (Figure 2).

Postmortem transcriptome analyses of the brain tissues from RTT patients suggest decreased transcript levels of synaptic vesicle proteins such as SNAP25 (Synaptosome- associated protein 25) in the frontal and parietal cortices [38]. SNAP25 is a protein element of the soluble N-ethylmaleimide-sensitive factor attachment protein receptor (SNARE), with crucial roles in the release of γ-aminobutyric acid (GABA) in stimulus-driven neurotransmission at the pre-synapse [39]. Impairments in the pre-synaptic protein parts of the GABAergic synapses are reported in the postnatal ventrolateral medulla of *Mecp2*-deficient mice [40]. As SNAP25 is important to neurotransmitter release at the pre-synapse, SNAP25 protein expression was examined. Our Western blot analyses indicated that SNAP25 was significantly increased (*p* < 0.01) in the cortex of the heterozygous female *Mecp2^T158M^* mice and the thalamus (*p* < 0.05) of the hemizygous male *Mecp2^T158M^* mice compared to these values in the wild-type controls (Figure 2). We also studied the expression of beta-3 tubulin (a neuronal marker) and Tau (a neuronal-microtubule-associated protein). We found that beta-3 tubulin was significantly reduced in the cortex of the heterozygous female *Mecp2^T158M^* mice compared to this value in the wild-type controls (** *p* < 0.01), but no significant change was observed for the Tau protein.

**Figure 3:**
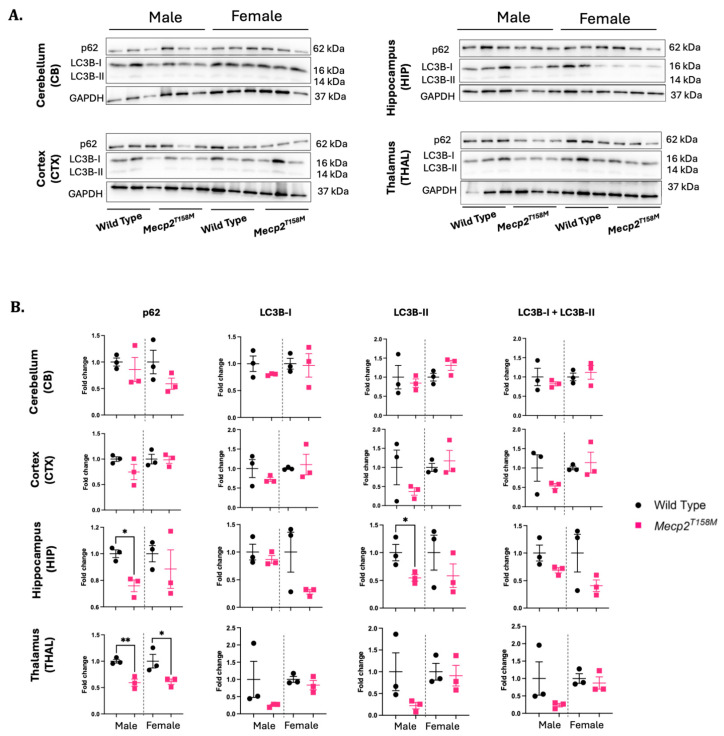
Expression levels of autophagy factors in the hemizygous male and heterozygous female *Mecp2^T158M^* mice and wild-type control mice. (**A**) The detection of p62 and LC3B-I/II through Western blot in the cerebellum (CB), cortex (CTX), hippocampus (HIP), and thalamus (THAL) of male and female *Mecp2^T158M^* mice and wild-type controls. (**B**) Quantification of signals performed through normalization of the relative band intensities with their corresponding GAPDH signals. Fold change calculated relative to the average wild-type fold change in the corresponding sex. Statistical analysis data were derived using an unpaired *t*-test and presented as the mean ± SEM (*n* = 3). The level of significance is shown as * *p* < 0.05 and ** *p* < 0.01. Uncropped images are shown in Supplementary Figure S4.

Postmortem analyses of RTT patients’ brains show large amounts of autophagy-like organelles, particularly in the cerebellar Purkinje cells [41]. Further, a proteomic analysis of the primary dermal fibroblasts from RTT patients indicated altered expression of mitophagy genes, along with an impaired mitophagy pathway [42]. Similar defective autophagic processing was observed in the retention of mitochondria in red blood cells from RTT patients [43]. A recent in vitro study reported defective autophagy in cortical *Mecp2*-deficient neurons isolated from male embryos. The authors found that *Mecp2*-deficient neurons show a significant decrease in the LC3B-II/LC3B-I ratio, with no significant difference in the p62 autophagic receptor, compared to these values in the controls [44]. To assess the effect of the MeCP2 T158M missense mutation on impaired autophagy in the brain, we performed Western blotting tests for the autophagy factors p62 and LC3 (I/II). Brain-region-dependent patterns of increased and decreased levels of protein expression were observed (Figure 3). Intriguingly, p62 was significantly reduced in the hippocampus and thalamus of the hemizygous male *Mecp2^T158M^* mice and the thalamus of heterozygous female *Mecp2^T158M^* mice compared to these values in the wild-type (Figure 3). Also, LC3B-II, which is a marker of autophagosomes, was significantly reduced (* *p* < 0.05) in the hippocampus of the hemizygous male *Mecp2^T158M^* mice compared to this value in the wild-type controls (Figure 3).

**Figure 4:**
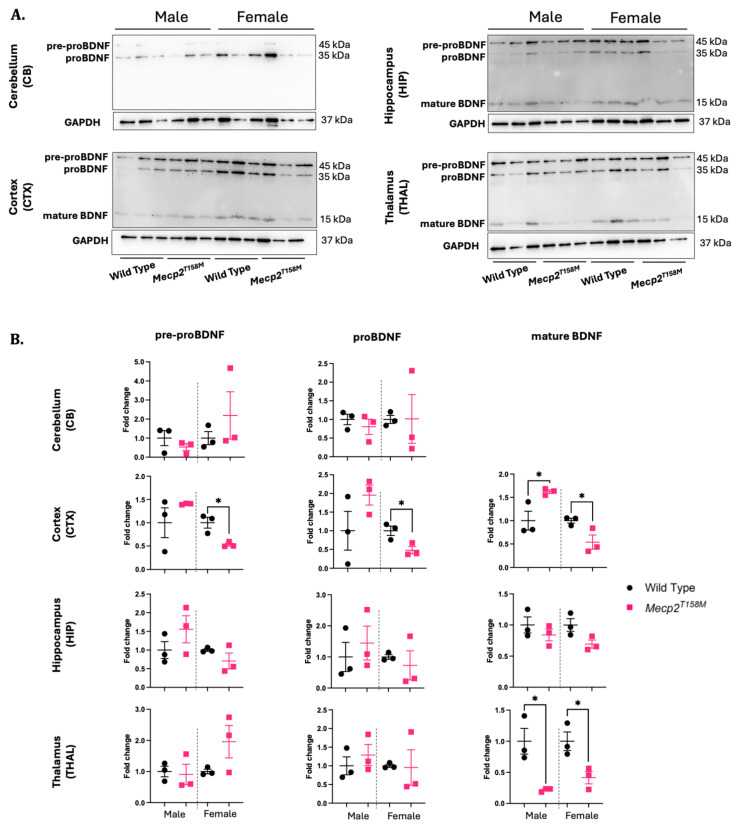
Expression levels of mature BDNF and BDNF precursors—pre-proBDNF and proBDNF—in the hemizygous male and heterozygous female *Mecp2^T158M^* mice and wild-type control mice. (**A**) The detection of pre-proBDNF, proBDNF, and mature BDNF through Western blot in the cerebellum (CB), cortex (CTX), hippocampus (HIP), and thalamus (THAL) of the hemizygous male and heterozygous female *Mecp2^T158M^* mice and wild-type controls. (**B**) Quantification of signals was completed by normalizing specific band intensities with their related GAPDH signals. Fold change analyzed compared to the average wild-type fold change in the corresponding sex. The statistical analysis was carried out using an unpaired *t*-test and the results presented as the mean ± SEM (*n* = 3). Significance of the data is shown as * *p* < 0.05. Uncropped images are shown in Supplementary Figure S5.

The functional interaction between BDNF and MeCP2 has been well established [29,45,46,47,48]. In postmortem brain tissue from RTT patients, the *BDNF* mRNA levels are reduced. However, Western blot analyses have indicated that the BDNF protein levels in brain tissue from RTT patients are not significantly changed postmortem, while IHC studies suggest elevated BDNF in the Purkinje cells of the cerebellum [29,45]. Meanwhile, in the cortex, the cerebellum, and the remainder of the brain in *Mecp2*-null mice (male mice aged six to eight weeks old), BDNF decreases by 21%, 41%, and 55%, respectively, and by 30% in the whole brain [49]. In our study, the Western blot analysis showed sex- and brain-region-specific trends towards an increase or decrease in the expression of BDNF and its precursors in the *Mecp2^T158M^* mice *versus* these values in the wild-type controls (Figure 4). Statistically lower levels of pre-proBDNF (* *p* < 0.05), proBDNF (* *p* < 0.05), and mature BDNF (* *p* < 0.05) were observed in the cortex of heterozygous female *Mecp2^T158M^* mice compared to those in the wild-type (Figure 4). Meanwhile, a statistically higher level of mature BDNF (* *p* < 0.05) was observed in the cortex of the hemizygous male *Mecp2^T158M^* mice (Figure 4). In the thalamus, a statistically lower level of mature BDNF (* *p* < 0.05) was observed in both the hemizygous male and heterozygous female *Mecp2^T158M^* mice compared to that in the wild-type controls (Figure 4).

### 3.3. The RNA Transcript Levels of the Mecp2e1 and Mecp2e2 Isoforms, as Well as Bdnf, in the Brains of Hemizygous Male Mecp2^T158M^ and Wild-Type Mice

**Figure 5:**
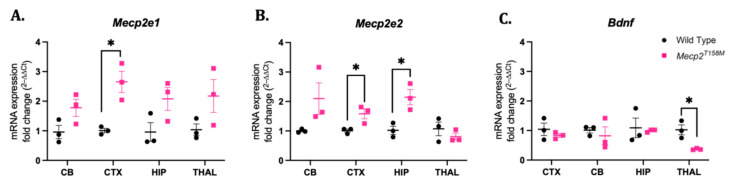
Transcript levels of *Mecp2e1*, *Mecp2e2*, and *Bdnf* in the brains of hemizygous male *Mecp2^T158M^* mice and wild-type control mice. (**A**) Quantified expression of *Mecp2e1* transcripts in the cerebellum (CB), cortex (CTX), hippocampus (HIP) and thalamus (THAL) of hemizygous male *Mecp2^T158M^* mice. (**B**,**C**) Quantified expression of the same *Mecp2e2* and *Bdnf* transcripts as those in (**A**), respectively. The fold change is analyzed relative to the average in the wild-type and reported as the fold change. The statistical analysis was carried out using an unpaired *t*-test and the results presented as the mean ± SEM (*n* = 3). The significance of the data is shown as * *p* < 0.05.

The *Mecp2*/*MECP2* gene is alternatively spliced to yield two protein isoforms, namely MeCP2E1 and MeCP2E2, which differ only by their N-terminal domains [50,51]. A previous study by our group has shown that in human brains, *MECP2E1* and *MECP2E2* transcript expression is brain-region-dependent [45]. Specifically, the expression of *MECP2E1* and/or *MECP2E2* was shown to be significantly lower in the amygdala, hippocampus, cerebrum, and cerebellum of female RTT patients with different *MECP2* mutations compared to that in controls. Additionally, the *MECP2E1* expression was significantly higher than the *MECP2E2* expression in all brain regions studied [45]. In the same study, we found that the *BDNF* transcript expression was significantly lower in the brains of female RTT patients compared to that in controls [45]. Therefore, as a means of investigating the effect of the T158M mutation on transcript expression, we measured the *Mecp2e1*, *Mecp2e2*, and *Bdnf* transcript levels in the brains of hemizygous male *Mecp2^T158M^* mice. Here, *Mecp2e1* expression was significantly increased in the cortex (* *p* < 0.05) while *Mecp2e2* expression was significantly increased in both the cortex (* *p* < 0.05) and the hippocampus (* *p* < 0.05) of the hemizygous male *Mecp2^T158M^* mice as compared to these values in the wild-type controls (Figure 5). Further, the *Bdnf* transcript expression in the male *Mecp2^T158M^* mice was not significantly different compared to that in the wild-type controls in all brain regions tested, except for the thalamus. Indeed, the *Bdnf* transcript expression was significantly lower in the thalamus (* *p* < 0.05) of the male *Mecp2^T158M^* mice compared to that in the wild-type controls.

### 3.4. Immunofluorescent Cellular Detection of Specific Proteins in the Brains of Male and Female Mecp2^T158M^ Mice

**Figure 6:**
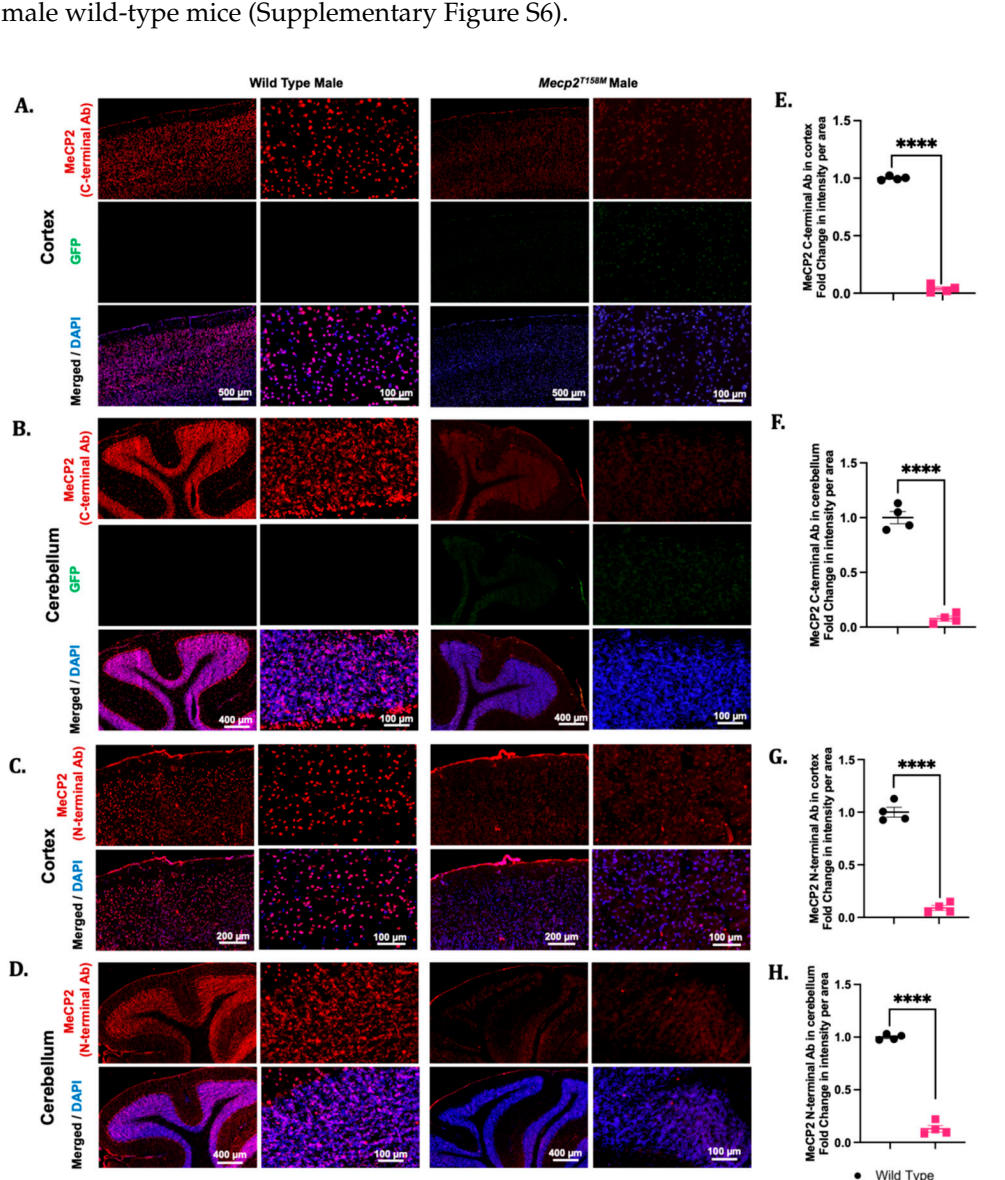
MeCP2 and green fluorescent protein (GFP) expression in the brains of hemizygous male *Mecp2^T158M^* mice and age-matched wild-type controls. The immunofluorescence assessment of the cortex and the cerebellum of wild-type male and hemizygous male *Mecp2^T158M^* mice (**A**–**D**), including the corresponding signal intensity measurements, shown as the mean fold change per area measured (**E**–**H**). In (**A**), the neocortex shows different cortical layers and the expression of MeCP2 (detection with an antibody against the C-terminal of MeCP2) (red), along with GFP, which is only expressed in the mutants, as well as the expression of MeCP2 (detection with an antibody against the N-terminal of MeCP2) (**C**). In (**B**,**D**), similar MeCP2 and GFP protein expression levels are seen among the arbor vitae of the cerebellum, showing the granular and molecular layers. DAPI signals are shown in blue. Scale bars (in µm) are shown in white font. Quantification of each IHC panel (**A**–**D**) is shown in the adjacent graph on the right side (**E**–**H**). The statistical analysis was performed using an unpaired *t*-test and the results presented as the mean ± SEM (*n* = 4). The significance of the data is shown as **** *p* < 0.0001. Primary antibody omission images are shown in Supplementary Figure S7.

We have previously reported the MeCP2 expression in different regions of the brain in both male and female adult mice [12,13]. Here, we observed significantly reduced MeCP2 expression in the cortex and cerebellum of the hemizygous male *Mecp2^T158M^* mice compared to that in the wild-type (Figure 1). Therefore, we examined the cellular distribution of MeCP2 in these regions using immunofluorescence. We used two anti-MeCP2 antibodies corresponding to the C- and N-terminal regions of the protein. We also examined the cellular distribution of GFP in the brains of hemizygous male *Mecp2^T158M^* mice. Signal intensity measurements confirm a significant reduction in both the cortex (**** *p* < 0.0001) and the cerebellum (**** *p* < 0.0001) of hemizygous male *Mecp2^T158M^* compared to these values in wild-type male mice. Here, we used antibodies against both the C- and N-terminal regions of MeCP2 (Figure 6). Similarly, the MeCP2 expression appeared to be reduced in the hippocampus and thalamus regions of the brains from the heterozygous female *Mecp2^T158M^* mice compared to that in the wild-type controls (Supplementary Figure S6). The MeCP2 expression was widely distributed in the hippocampus and thalamus of the male wild-type mice (Supplementary Figure S6).

**Figure 7:**
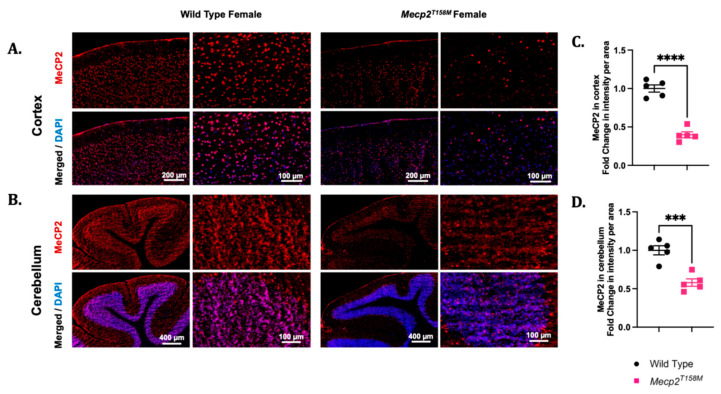
The MeCP2 expression in the brains of heterozygous female *Mecp2^T158M^* mice and age-matched wild-type controls. Immunofluorescent assessment of the cortex and the cerebellum of wild-type female and heterozygous female *Mecp2^T158M^* mice (**A**,**B**), including the corresponding signal intensity measurements, shown as the mean fold change per area measured (**C**,**D**). In (**A**), the cortex shows different cortical layers and the expression of MeCP2 (detection with an antibody against the N-terminal of MeCP2) (red). In (**B**), similar MeCP2 protein expression levels are seen among the arbor vitae of the cerebellum, showing the granular and molecular layers. DAPI signals are shown in blue. Scale bars (in µm) are shown in white font. Quantification of each IHC panel (**A**,**B**) is shown in the adjacent graph on the right side (**C**,**D**). The statistical analysis was carried out using an unpaired *t*-test and presented as the mean ± SEM (*n* = 5). The significance of the data is shown as *** *p* < 0.001 and **** *p* < 0.0001. Primary antibody omission images are shown in Supplementary Figure S9.

We also observed alterations in the MeCP2 expression in the heterozygous female *Mecp2^T158M^* mice compared to that in the wild-type in both the cortex and cerebellum through the Western blot analysis (Figure 1). Thus, we examined immunofluorescent detection of MeCP2 in these regions using IHC in the heterozygous *Mecp2^T158M^* female mice and wild-type controls. Signal intensity measurements suggested a significant reduction in the MeCP2 levels in the cortex of the female heterozygous *Mecp2^T158M^* mice *versus* that in the wild-type controls (**** *p* < 0.0001) (Figure 7)—a result similarly observed in the Western blot analysis of the same brain region (Figure 1). While our Western blot analyses indicated no significant difference in the MeCP2 expression in the cerebellum of the heterozygous female *Mecp2^T158M^* mice, signal intensity measurements of the IHC images using the N-terminal anti-MeCP2 antibody suggested a significant reduction in MeCP2 expression levels (*** *p* < 0.001) (Figure 7). A wide distribution of MeCP2 was also observed in the hippocampus and thalamus of the female wild-type mice through IHC (Supplementary Figure S7). Meanwhile, the MeCP2 expression was weakly detected in the same brain regions of the heterozygous female *Mecp2^T158M^* mice when compared to that in the wild-type (Supplementary Figure S7).

**Figure 8:**
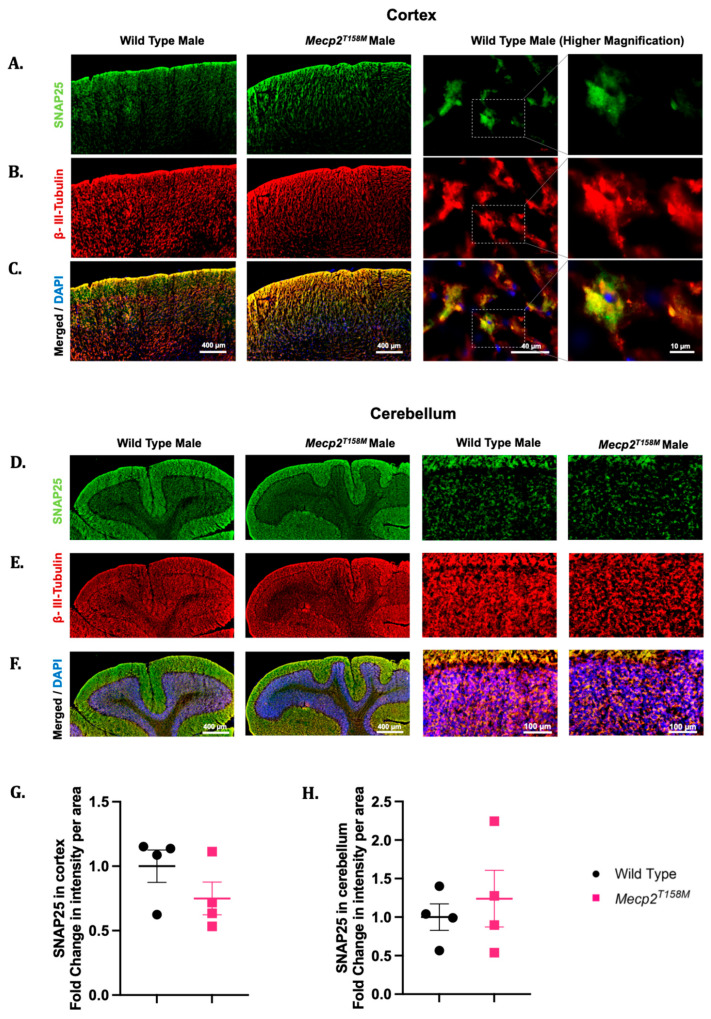
The SNAP25 and β-III-tubulin expression in the brains of hemizygous male *Mecp2^T158M^* mice and wild-type controls. Immunofluorescent assessment of the cortex and cerebellum of wild-type male and hemizygous male *Mecp2^T158M^* (**A**–**F**), including the corresponding signal intensity measurements, shown as the mean fold change per area measured (**G**,**H**). Here, SNAP25 is shown in green, β-tubulin III is shown in red, and DAPI signals are shown in blue. Scale bars (in µm) are shown in white font. The statistical analysis was carried out using an unpaired *t*-test and the results presented as the mean ± SEM (*n* = 4). Primary antibody omission images are found in Supplementary Figure S7.

As indicated earlier, our Western blot analyses showed changes in the SNAP25 expression in certain brain regions of the hemizygous male *Mecp2^T158M^* mice (Figure 2). The Western blot analysis showed no significant difference in the SNAP25 expression in the cerebellum and the cortex of the hemizygous male *Mecp2^T158M^* mice when compared to that in the wild-type controls. Specifically, a slight trend of an increase in SNAP25 expression was observed in the cerebellum while a slight trend of a decrease in SNAP25 expression was evident in the cortex in the Western blot analysis. Similar trends in SNAP25 expression were observed via IHC when the signal intensity was measured in the cerebellum and cortex of the hemizygous male *Mecp2^T158M^* mice (Figure 8A–H).

**Figure 9:**
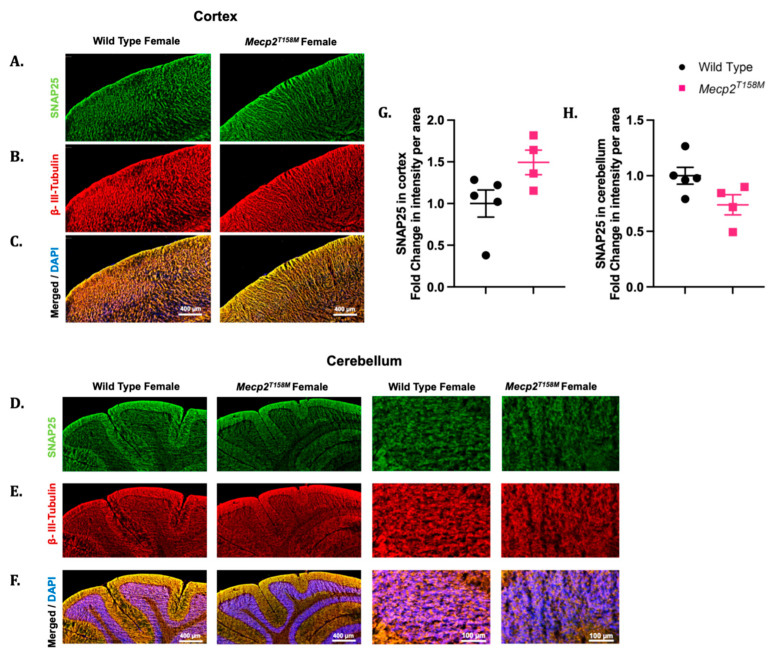
The SNAP25 and β-III-tubulin expression in the brains of heterozygous female *Mecp2^T158M^* mice and wild-type controls. Immunofluorescent assessment of the cortex and cerebellum of wild-type female and heterozygous female *Mecp2^T158M^* mice (**A**–**F**), including the corresponding signal intensity measurements, shown as the mean fold change per area measured (**G**,**H**). Here, SNAP25 is shown in green, β-tubulin III is shown in red, and DAPI signals are shown in blue. Scale bars (in µm) are shown in white font and in µm. The statistical analysis was carried out using an unpaired *t*-test and the results presented as the mean ± SEM (*n* = 4–5). Primary antibody omission images are found in Supplementary Figure S9.

We then evaluated the SNAP25 protein expression in the cortex and cerebellum of the heterozygous female *Mecp2^T158M^* mice through IHC. Although the Western blot analyses suggested a significant increase in the SNAP25 protein expression in the cortex of the heterozygous female *Mecp2^T158M^* mice (Figure 2), similar statistically significant results were not evident in the IHC assessments, despite the presence of an increasing trend (Figure 9). However, both the Western blot (Figure 2) and IHC assessments demonstrated a decreasing trend in the SNAP25 expression levels in the cerebellum of the heterozygous female *Mecp2^T158M^* mice as compared to those in the wild-type controls, which was not statistically significant (Figure 9).

### 3.5. Phenotypic Characterization of the Mecp2^T158M^ Mice Indicates Sex-Specific Motor Control Impairments in Hemizygous Males and an Increased Body Weight in Heterozygous Females Compared to Wild-Type Mice

**Figure 10:**
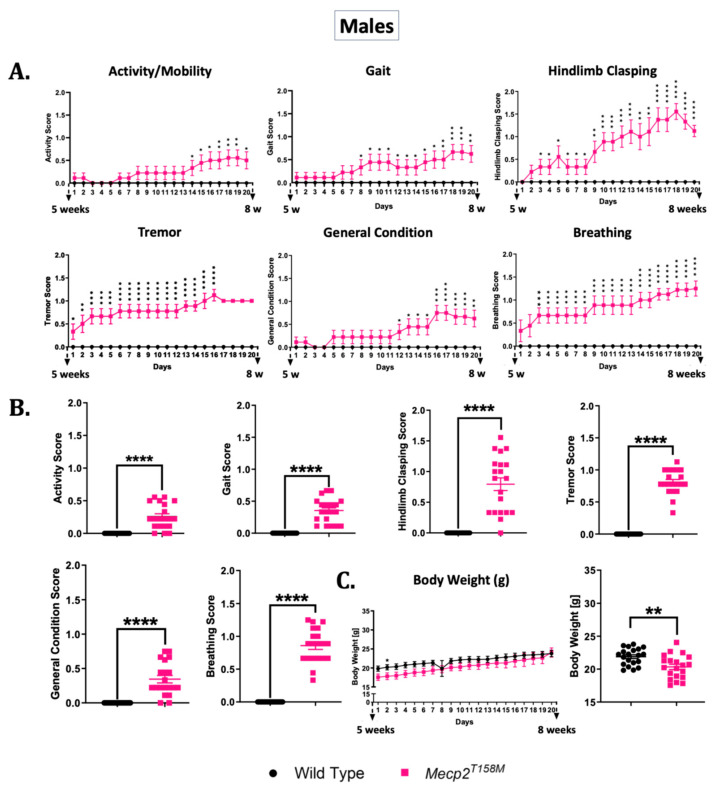
The phenotypic assessment of hemizygous male *Mecp2^T158M^* mice compared to wild-type controls. Hemizygous male mice (*Mecp2^T158M^*) and wild-type controls were monitored daily from approximately 5 weeks to 8 weeks of age (20 days). Six phenotypic parameters—activity/mobility, gait, hindlimb clasping, tremor, general condition (appearance), and breathing—were scored daily and are presented in two formats: (**A**) line graphs, showing the mean ± SEM for each group on each day (*n* = 10 wild-type and *n* = 9 *Mecp2^T158M^* mice), and (**B**) bar graphs, showing the overall mean scores over the course of 20 days. (**C**) Body weight (in grams) is shown in a similar format to that in (**A**,**B**). For the bar graphs, each data point represents the mean daily score for all mice on that day (i.e., 20 time points per group for the 20 days), not for individual mice. The statistical analysis for both formats was performed using unpaired *t*-tests, and the results are shown as mean ± SEM. Statistical significance is shown as: * *p* < 0.05; ** *p* < 0.01; *** *p* < 0.001; **** *p* < 0.0001.

The phenotypic assessment of the *Mecp2^T158M^* mice involved a series of observational tests commonly used for RTT mice [9]. These tests were designed to capture the spectrum of RTT-like symptoms, including motor dysfunction, respiratory abnormalities, tremor, and general health deficits. Studies suggest that RTT mouse models recapitulate a wide range of the symptoms of human RTT patients. Particularly, both human RTT patients and RTT mouse models experience seizures [52,53] and breathing irregularities [54,55]. Thus, we utilized a scoring system involving daily observations of weight and six specific symptoms for a period of 20 days during the timeline of the onset of symptoms in the male and female RTT mice. This included scoring of the gait, hindlimb clasping, activity/mobility, general condition, tremor, and breathing (Supplementary Table S1). Each scoring criterion was given a number on a scale of 0 to 2, where 0 indicated that the symptom was absent, 1 indicated that it was present, and 2 indicated that it was severe. The wild-type mice consistently scored zero across all categories, serving as a baseline for comparison [9]. The hemizygous male *Mecp2^T158M^* mice exhibited a variety of RTT-like symptoms, including a lower body weight, reduced activity, abnormal gait, moderate hindlimb clasping, a poor general condition (appearance), tremor, and rapid or irregular breathing in both sexes, evident as increase in the scoring values. The daily scoring system facilitated tracking of the phenotypic abnormalities and symptomatic progression in the mutant mice (Figure 10).

As illustrated in Figure 10, all six phenotypic metrics demonstrated a statistically significant change in scores for the hemizygous male *Mecp2^T158M^* mice relative to those in the wild-type control mice. Over the course of 20 days, the phenotypic scores of the hemizygous male *Mecp2^T158M^* mice worsened. All criteria (activity/mobility (**** *p* < 0.0001), gait (**** *p* < 0.0001), hindlimb clasping (**** *p* < 0.0001), tremor (**** *p* < 0.0001), general condition (**** *p* < 0.0001) and breathing (**** *p* < 0.0001)) had a significant overall increase in the *Mecp2^T158M^* mice relative to those in the wild-type controls. Concurrently, the mutant male mice exhibited a significant reduction (** *p* < 0.01) in their overall body weight compared to that of their wild-type counterparts; however, they did continue to gain weight like their wild-type counterparts.

**Figure 11:**
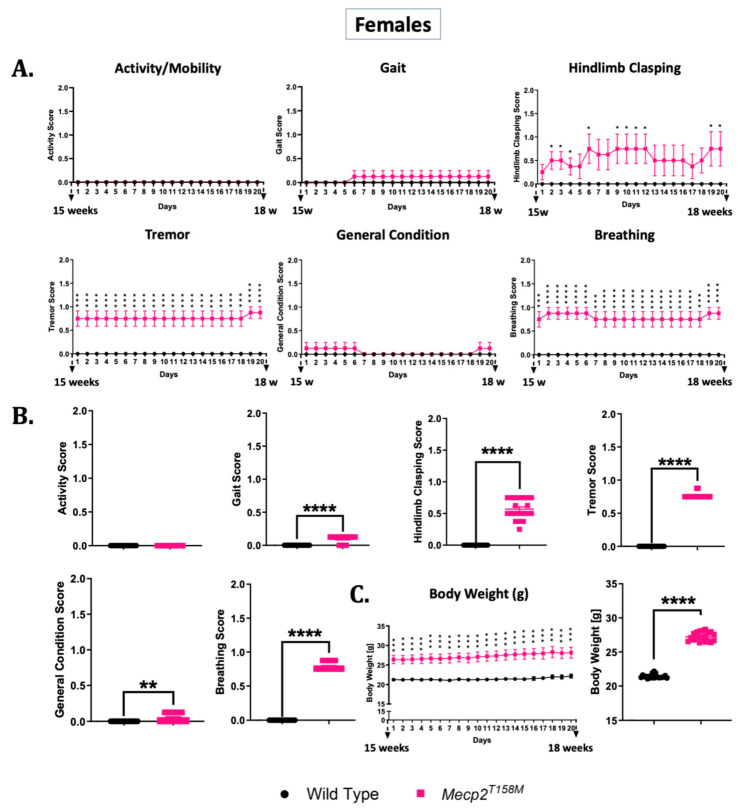
The phenotypic assessment of heterozygous female *Mecp2^T158M^* mice compared to wild-type controls. Heterozygous female mice (*Mecp2^T158M^*) and wild-type controls were monitored daily from approximately 15 weeks to 18 weeks of age (20 days). Six phenotypic parameters—activity/mobility, gait, hindlimb clasping, tremor, general condition (appearance), and breathing—were scored daily and are presented in two formats: (**A**) line graphs, showing the mean ± SEM for each group on each day (*n* = 10 wild-type and *n* = 8 *Mecp2^T158M^* mice), and (**B**) bar graphs, showing the overall mean scores over the course of the 20 days. (**C**) Body weight (in grams) is shown in a similar format to that in (**A**,**B**). For the bar graphs, each data point represents the mean daily score for all mice on that day (i.e., 20 time points per group for the 20 days), not for individual mice. Both formats of analysis were performed using unpaired *t*-tests, and the results are shown as the mean ± SEM. Statistical significance is shown as: * *p* < 0.05; ** *p* < 0.01; *** *p* < 0.001; **** *p* < 0.0001.

As illustrated in Figure 11, five of the six phenotypic parameters (gait (**** *p* < 0.0001), hindlimb clasping (**** *p* < 0.0001), tremor (**** *p* < 0.0001), general condition (** *p* < 0.01), and breathing (**** *p* < 0.0001)) significantly worsened in the *Mecp2^T158M^* mice relative to the wild-type controls. Activity, gait, tremor, and breathing scores remained generally stable over the course of 20 days, while the hindlimb clasping score significance varied over the course of the 20 days. Overall body weight was also significantly higher (**** *p* < 0.0001) in the female *Mecp2^T158M^* mice relative to that in the wild-type controls. Over the course of 20 days, the body weight of the female *Mecp2^T158M^* mice appeared to increase, while the body weight of the wild-type control females remained relatively stable.

**Figure 12:**
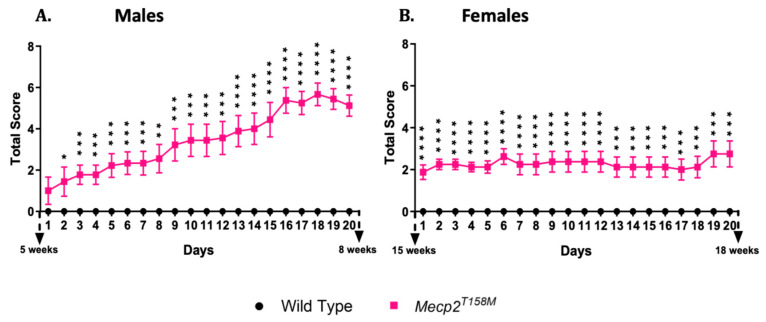
Total phenotypic scores of hemizygous male and heterozygous female *Mecp2^T158M^* mice compared to those in wild-type controls. For hemizygous male mice (**A**) and heterozygous female mice (**B**), the presented total score is the cumulative summary of all six phenotypic parameters—activity/mobility, gait, hindlimb clasping, tremor, general condition (appearance), and breathing—and is displayed as a line graph showing each of the 20 days for each sex. The *y*-axis represents the score (higher score = worsened condition). A statistical analysis was performed using an unpaired *t*-test and the results presented as the mean ± SEM, with *n* = 10 for male wild-type, *n* = 9 for male *Mecp2^T158M^*, *n* = 10 for female wild-type and *n* = 8 for female *Mecp2^T158M^* mice. The levels of significance are shown as * *p* < 0.05, ** *p* < 0.01, *** *p* < 0.001, **** *p* < 0.0001.

The total score, which reflects the sum of all six phenotypic scores, serves as a comprehensive indicator of the cumulative effects. As shown in Figure 12, in the *Mecp2^T158M^* mice, both hemizygous males and heterozygous females display significant worsening of their scores (* *p* < 0.05, ** *p* < 0.01, *** *p* < 0.001, **** *p* < 0.0001) compared to those in the wild-type mice. Over the course of 20 days of scoring, the male *Mecp2^T158M^* mice scored significantly higher, while the female *Mecp2^T158M^* mice began with significantly higher scores, which then remained relatively stable. This demonstrates the severity of the T158M mutation and may highlight notable differences between sexes.

### 3.6. Behavioural Testing in Mecp2^T158M^ Mice Suggests Sex-Specific Effects of the MeCP2 T158M Mutation on Anxiety-like Behaviour and Motor Function

**Figure 13:**
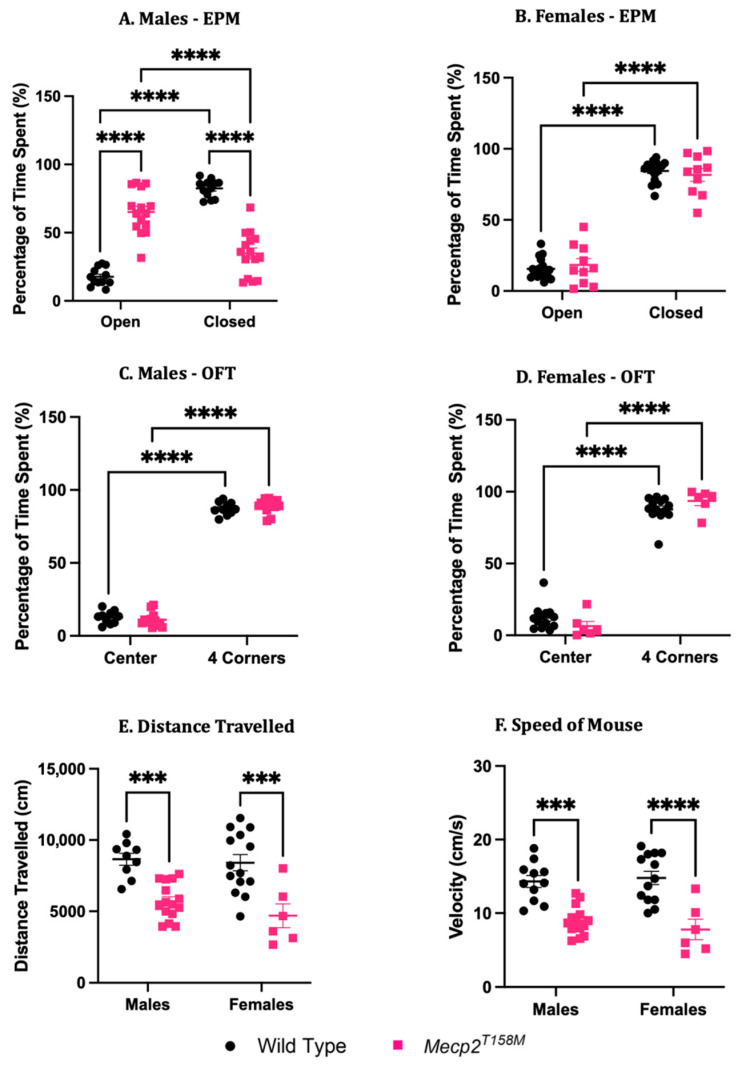
A comparison of wild-type and *Mecp2^T158M^* males and females in terms of their anxiety-like behaviours and locomotion. (**A**,**B**) The percentage of time that mice spent in the closed and open arms of the elevated plus maze (EPM). Mice were placed at the centre of the maze and allowed to explore different arms for a total of 10 min. (**C**,**D**) The percentage of time spent in the centre, as well as in all four corners of the box. Mice were placed at the centre of the box and were permitted to explore the areas for a total of 10 min (600 s). (**E**,**F**) Total distance traveled (in cm), as well as the speed of the mouse, in the open field test (OFT). For the EPM, the number of mice per group includes male wild-type (*n* = 12), male hemizygous *Mecp2^T158M^* (*n* = 15), female wild-type (*n* = 17), and female heterozygous *Mecp2^T158M^* (*n* = 10) mice. For the OFT (time spent), the number of mice per group includes male wild-type (*n* = 10), male hemizygous *Mecp2^T158M^* (*n* = 13), female wild-type (*n* = 13), and female heterozygous *Mecp2^T158M^* (*n* = 6) mice. For the OFT (speed), the number of mice per group includes male wild-type (*n* = 11), male hemizygous *Mecp2^T158M^* (*n* = 14), female wild-type (*n* = 13), and female heterozygous *Mecp2^T158M^* (*n* = 6) mice. For the OFT (distance), the number of mice per group includes male wild-type (*n* = 9), male hemizygous *Mecp2^T158M^* (*n* = 15), female wild-type (*n* = 14), and female heterozygous *Mecp2^T158M^* (*n* = 6) mice. The results are shown as the mean ± SEM. The data was analyzed through a two-way ANOVA with Tukey’s multiple comparisons test; *** *p* < 0.001 and **** *p* < 0.0001.

Among the spectrum of neurological complications experienced by RTT patients, change in anxiety levels is a prominent characteristic [56]. Studies suggest that mouse models of RTT experience a similar impact on mobility and the development of anxiety-like behaviour similar to clinical symptoms manifested in human RTT patients [57,58,59]. Thus, we performed behavioural tests to examine the hemizygous male and heterozygous female *Mecp2^T158M^* mice and compared them to age-matched wild-type controls. The elevated plus maze was used to study anxiety-like behaviours in the male and female mice, where the percentage of time spent in the open arms *versus* closed arms was calculated. Mice that spend more time in the closed arms than in the open arms typically represent normal anxiety levels [20,21]. In the EPM test, the time the wild-type male mice spent in the open arms was significantly lower compared to that spent in the closed arms (*** *p* < 0.001). The hemizygous *Mecp2^T158M^* male mice showed the opposite behaviour, as they spent more time in the open arms, which was significantly higher compared to that in the closed arms (*** *p* < 0.001) (Figure 13A and Supplementary Figure S10A,B). This suggests greatly reduced anxiety in the hemizygous male *Mecp2^T158M^* mice. In contrast, both the female wild-type and heterozygous female *Mecp2^T158M^* mice spent less time in the open arms compared to the closed arms, and this was statistically significant (**** *p* < 0.0001) (Figure 13B and Supplementary Figure S10A,B), with no significant differences observed between them.

The open field test was used primarily to assess locomotion (distance travelled and velocity), as well as exploratory behaviour (the percentage of time spent in the centre part *versus* all four corners) [21,22]. In the open field test, the percentage of time spent in the open part *versus* the four corners was significantly less in both the wild-type and hemizygous *Mecp2^T158M^* male mice (**** *p* < 0.0001; Figure 13C). The same was observed in the females (**** *p* < 0.0001; Figure 13D). However, the distance travelled was significantly reduced for the hemizygous male (*** *p* < 0.001) and heterozygous female (*** *p* < 0.001) *Mecp2^T158M^* mice compared to that for the wild-type controls, where the same was observed for velocity, suggesting reduced motor coordination overall in both sexes (Figure 13E,F). However, when comparing the sexes, there were no significant differences between males and females, despite their age differences (Figure 13E,F). In the OFT tracking maps (Supplementary Figure S11B), a straight-line pattern was observed for the wild-type males and females, but a squiggle-like pattern was observed among the *Mecp2^T158M^* mice of both sexes, suggesting a potential gait issue.

## 4. Discussion

MeCP2 plays an essential role during the development of the mammalian brain, from the late embryonic stages into adulthood [60]. Elucidation of the downstream ramifications of mutations in *MECP2* in relation to Rett Syndrome and other neurodevelopmental diseases could provide alternative therapeutic avenues. In this study, the gross brain features and molecular impact of the MeCP2 T158M missense mutation were explored in mice to further characterize the impact of this frequently occurring RTT-associated MeCP2 mutation. The results of the present study confirm that the T158M mutation reduces MeCP2 levels in the brain, affecting the brain’s weight and size, and the protein expression in the brain—findings that may relate to the pathology of RTT in human patients [60,61].

RTT’s neuropathology is largely underscored by the smaller weight and size of the brain [61], a finding that is recapitulated in the *Mecp2^T158M^* mouse model used here. The expression of MeCP2 in neurodevelopment begins in the early stages of embryonic development and substantially increases in later stages [13,62]. During development, MeCP2 regulates the expression of genes related to neuronal maturation and synapse maturation, including *BDNF*, early growth response protein 2 (*EGR2*), inhibitor of DNA binding 1 (*ID1*), and JunB Proto-Oncogene (*JUNB*) [62,63,64]. It is important to note that while RTT patients have a reduced brain weight, there is no reported loss of brain cells, suggesting that the RTT-affected brain does not show signs of degeneration or atrophy. Instead, the density or packing of the cells in the RTT brain is much greater than normal [65]. Thus, MeCP2 mutations, as in the case of RTT, may directly relate to stagnated brain growth.

The T158M missense mutation in the methyl binding domain of MeCP2 disrupts vital hydrogen bonding, which maintains the stability of the ASX turn (Asp156-Phe157-Thr158) and the ST motif (Thr158-Val159-Thr160-Gly161) [32]. Ultimately, the T158M mutation in MeCP2 yields an unstable MeCP2 product. It is not surprising that our Western blot analysis showed reduced MeCP2 levels in both the hemizygous male and heterozygous female *Mecp2^T158M^* mice compared to those in the wild-type controls. The reduction in MeCP2 levels in the *Mecp2^T158M^* mice may also be explained by possible protein degradation pathways. For instance, previous studies suggest that unstable MeCP2 T158M is subjected to degradation via the ubiquitin–proteasome pathway [3], with an isoform-specific auto-regulatory mechanism involving the proteosome pathway [23]. Here, our results did not show an increase in MeCP2 levels in response to the detected increase in *Mecp2e1*/*e2* transcript levels that we observed in specific brain regions (the cortex for *Mecp2e1* and the cortex and hippocampus for *Mecp2e2*) (Figure 5). However, it is important to note that there are multiple regulatory steps that control the translation of a specific protein from the corresponding transcripts [66]. It is possible that the increase in *Mecp2e1*/*e2* transcripts may be a form of overcompensation following the degradation of the unstable MeCP2 T158M protein product. In a previous study of postmortem brain tissues from human RTT patients, the transcript levels of *MECP2* isoforms were significantly reduced in specific brain regions. However, the detected protein levels were not significantly different from those in control non-Rett Syndrome brain tissues [45].

The impact of MeCP2 deficiency is neurologically widespread and may begin early in embryonic development. Much of embryonic and post-embryonic mammalian brain development relies on MeCP2-driven events. Therefore, it can be speculated that the aberrant expression of MeCP2 influences these key neurodevelopmental events. An increase in MeCP2 during neurodevelopment coincides with an increase in *BDNF* levels [67]. MeCP2 transcriptionally regulates the *BDNF*/*Bdnf* expression in response to neuronal activity (firing of action potentials). Binding of MeCP2 to the *BDNF*/*Bdnf* regulatory regions represses transcription. This repression is relieved following neuronal activity and the subsequent release of MeCP2 from the *BDNF*/*Bdnf* promoter [48]. Typically, BDNF is synthesized as the pre-proBDNF precursor. Subsequently, proBDNF is formed following the pre-sequence cleavage of pre-proBDNF [68]. Further cleavage of proBDNF results in the formation of mature BDNF [68]. While proBDNF function has been linked to long-term depression (a process which weakens the synaptic connections), mature BDNF is involved in promoting dendritic spine density and both synaptic maturation and plasticity—processes believed to relate to memory and learning [69]. In RTT, studies have underscored impaired BDNF trafficking and the activity-dependent release of BDNF in disease progression [70,71,72]. Additionally, lower amounts of BDNF at both the transcript and protein levels have been found in the context of RTT [49]. Region-specific dysregulation of BDNF protein levels has been reported in both RTT human and mouse model brains [73]. Thus, it was not surprising that MeCP2-deficient mice used in this study displayed altered BDNF expression. Given the role of proBDNF in long-term depression, altered proBDNF expression may contribute to impairments in the brain’s synaptic plasticity in RTT patients and mouse models. Further, as BDNF is involved in pathways that support neuronal health, modulate synaptic strength, and facilitate learning and memory, altered expression of BDNF may negatively impact the developing brain [74].

One phenomenon particularly impaired by altered BDNF expression is synaptic plasticity, with activity-dependent changes in the strength of the neuronal connections [75]. Morphological, functional, and molecular alterations in the synapses *due to* altered BDNF expression in the context of RTT may manifest as a variety of clinical symptoms. Dysregulated BDNF expression, for instance, may promote seizures [76]. PSD95 is one key player in the maturation and maintenance of the synapses [77]. Through the PI3-Akt pathway, BDNF promotes the delivery of PSD95 to the synapses [78]. Meanwhile, BDNF-TrkB signaling promotes the palmitoylation of PSD95 and its subsequent delivery to the synapses [79]. Thus, significantly reduced levels of PSD95, especially in the thalamus, of MeCP2-deficent mice may be a downstream effect of both reduced MeCP2 and BDNF levels. Importantly, PSD95 is also responsible for the insertion of the ionotropic glutamate excitatory receptors AMPA and NMDA neurotransmitter receptors at the synaptic button. Additionally, PSD95 transgenic mouse models indicate that PSD95 deficiency promotes GABAergic-inhibitory synapse activity [80]. Altered PSD95 expression therefore may contribute to the excitatory/inhibitory (E/I) imbalance in the neurons observed in RTT patients and mouse models.

Changes in the pre-synaptic proteins involved in neurotransmitter release elucidate the synaptopathy of RTT. SNAP25, a component of the SNARE complex, aids in synaptic transmission via its role in exocytosis of the synaptic vesicles [81]. Thus, alterations in SNAP25 expression may lead to inefficient neurotransmitter release from the excitatory and/or inhibitory neurons. Significantly elevated extracellular levels of glutamate have been found in the cerebral cortex of glutamatergic-neuron-specific *Snap25* knockout mouse models [82]. Similarly, the findings of using *Snap25*-null mouse models reveal that GABAergic neuronal function depends on the SNARE complex and that SNAP25 is vital for GABA release [39]. Collectively, these findings suggest that in cases where SNAP25 expression is altered, there is potential for an imbalance in the release of excitatory and inhibitory neurotransmitters. This imbalance may translate into a plethora of symptoms in RTT patients and mouse models.

Post-mitotic neurons rely on neuronal autophagy for neuronal development, survival, aging, and death [83]. In comparison to proliferating cells, post-mitotic neurons are more susceptible to the accumulation of cytotoxic proteins [84]. Thus, impairments in neuronal autophagy underlie the neuropathology of other neurological and/or neurodevelopmental diseases including Parkinson’s disease, Alzheimer’s disease, Fragile X Syndrome, Vici syndrome, autism spectrum disorders, amyotrophic lateral sclerosis, Huntington’s disease, and Lafora disease [83]. Here, LC3B-II, a marker of autophagosomes and autophagic activity, was significantly reduced in a brain-region-specific manner in male RTT mouse models. Similarly, Esposito et al. found reduced levels of LC3B-II in mouse neuronal cultures and cortices, as well as in human RTT fibroblasts [44]. In line with these findings, the researchers also found a reduced amount of autophagic structures in neurons from a *Mecp2*-deficient knockout model, a hint towards possible impairment of autophagosome biogenesis [44].

As previously discussed, the molecular findings of this study converge in terms of the development and maintenance of the synapses in the brain. Synaptic alterations in the brains of RTT patients have been well established as a hallmark of disease progression in RTT [85,86,87]. While proteins such as BDNF, PSD95, and SNAP25 may be involved in the development of synaptic plasticity, autophagy factors such as p62 and LC3B I/II may be involved in synaptic pruning and the maintenance of synaptic plasticity [88]. These findings may further explain the phenotypic and behavioural findings of this study. Specifically, impairments in molecular pathways related to synaptic plasticity may underscore the deficits in learning and mobility in both RTT patients and mouse models. For instance, the abnormalities in hindlimb clasping observed in RTT mouse models may be related to the loss of purposeful hand movements in RTT patients. Thus, our findings suggest that the phenotypic and behavioural abnormalities observed in the present study may be elucidated by molecular abnormalities specific to synaptic pathways.

While brain-region-dependent perturbations in protein expression and behavioural abnormalities appear to be sex-dependent, X-chromosome inactivation (XCI) skewing in female mice adds another level of complexity. Female heterozygous RTT mice, like human RTT patients, are mosaic carriers of both normal and mutated copies *Mecp2*/*MECP2* [89]. As a result, the presentation of RTT in both mice and humans relies on organism-specific skewing of which copy of *Mecp2*/*MECP2* is expressed. Meanwhile, the absence of XCI skewing in hemizygous male RTT mouse models allows for a more direct investigation of the effect of reduced *Mecp2* levels. Further, the heterozygosity of female RTT mice and patients allows for one normal copy of *Mecp2*/*MECP2* to be expressed. Therefore, the presence of normal MeCP2 in females may compensate for the effect of the mutated MeCP2. Additionally, the sex-specific differences observed in this study may be the consequence of full MeCP2 loss in the hemizygous male mice and partial MeCP2 loss in the heterozygous female mice, rather than intrinsic sex differences. Nonetheless, male and female RTT mouse models closely recapitulate the clinical progression and symptoms commonly associated with RTT patients. However, our conclusions (at the protein levels detected through Western blotting) are limited to using *n* = 3 biological replicates per group. While additional biological replicates may increase the statistical power and reinforce the robustness of our conclusions, this sample size is consistent with common practices in molecular biology, where similar replicate numbers are frequently employed to demonstrate reproducible trends by independent groups [44,73,90]. We did, however, use 4–5 additional biological replicates in IHC studies to complement our Western blot experiments.

Clinically, RTT patients present with a variety of symptoms that may be related to impairments in specific brain regions. However, the heterogeneity of brain regions in terms of their distribution of cell populations (neurons, glial cells of non-neuronal origin) may translate into differences in cell-type-specific MeCP2 gene regulation. This heterogeneity may also explain the brain-region-specific results of this study. Thus, further investigation is required to determine which cell types particularly contribute to the presented results. Studies have accounted for the RTT-specific excitatory/inhibitory imbalance in the neurons of the amygdala, brainstem, cerebral cortex, and hippocampus in both human patients and mouse models [91,92,93,94]. Consequently, the excitatory/inhibitory imbalance observed in RTT may be a neuropathological effect that underlies many of its clinical and molecular features regardless of the brain region.

## 5. Conclusions

Here, we investigated the impact of the MeCP2 T158M mutation in a transgenic RTT mouse model. Our data suggested that the MeCP2 T158M mutation leads to a reduced weight and length of the brain in both male and female *Mecp2^T158M^* mice as compared to those in wild-type controls. We also observed brain-region- and sex-specific alterations in the expression profiles of MeCP2, as well as specific synaptic and autophagy proteins, in the male and/or female *Mecp2^T158M^* mice compared to age- and sex-matched wild-type controls. In this study, hemizygous male *Mecp2^T158M^* mice displayed motor control impairments, a reduced body weight, and a wide spectrum of phenotypic abnormalities compared to wild-type controls. Meanwhile, we observed an increased body weight as well as phenotypic impairments in certain phenotypic criteria in the heterozygous *Mecp2^T158M^* females compared to the wild-type controls. Further, our results suggested that sex-specific effects on anxiety-like behaviour and motor function are detectable in mutated *Mecp2^T158M^* mice. Collectively, our results highlight the sex-specific molecular, phenotypic, and behavioural impact of the MeCP2 T158M mutation in this transgenic RTT mouse model.

## Data Availability

All data regarding this manuscript exists within the manuscript (the main article and Supplementary Materials).

## Supplementary Materials

The following supporting information can be downloaded at https://www.mdpi.com/article/10.3390/cells14161286/s1, Figure S1. Comparative analysis of the mean brain weight (mg) and length (mm) of male and female *Mecp2^T158M^* mice and their age- and sex-matched wild-type controls; Figure S2. Uncropped images of Western blot signals for Figure 1; Figure S3. Uncropped images of Western blot signals for Figure 2; Figure S4. Uncropped images of Western blot signals for Figure 3; Figure S5. Uncropped images of Western blot signals for Figure 4; Figure S6. MeCP2 expression in the brain of male *Mecp2^T158M^* mice and wild-type controls; Figure S7. MeCP2 expression in the brain of female *Mecp2^T158M^* mice and wild-type controls; Figure S8. Controls verifying specificity of immunolabelling in the brain of male *Mecp2^T158M^* mice and wild-type controls; Figure S9. Controls verifying specificity of immunolabelling in the brain of female *Mecp2^T158M^* mice and wild-type controls; Figure S10. Heat and tracking maps for the elevated plus maze (EPM) for wild-type and *Mecp2^T158M^* males and females; Figure S11. Heat and track visualization maps for the open field test (OFT) for wild-type and *Mecp2^T158M^* males and females. Table S1. Scoring reference and endpoint monitoring checklist; Table S2. Antibodies used for Western blot and immunohistochemistry experiments.

## Abbreviations

The following abbreviations are used in this manuscript:

AMPA	α-amino-3-hydroxy-5-methyl-4-isoxazolepropionic acid
BDNF/*Bdnf*	Brain-derived neurotrophic factor
CACS	Central Animal Care Services
CB	Cerebellum
CTX	Cortex
*EGR2*	Early growth response protein 2
EHSO	Environmental Health and Safety Office
EPM	Elevated plus maze
GABA	γ-aminobutyric acid
GAPDH	Glyceraldehyde-3-phosphate dehydrogenase
GFP	Green fluorescent protein
GMC	University of Manitoba Genetic Modelling Centre
HIP	Hippocampus
ID1	Inhibitor of DNA binding 1
IHC	Immunohistochemistry
JUNB	JunB Proto-Oncogene
LC3BI/II	Microtubule- associated protein 1 light chain I/II
MeCP2	Methyl-CpG- binding protein 2 (protein)
*Mecp2*	Methyl-CpG- binding protein 2 (mouse gene)
*MECP2*	Methyl-CpG- binding protein 2 (human gene)
NMDA	N-methyl-D-aspartate
OFT	Open field test
p62	Sequestosome 1
PBS	Phosphate- buffered saline
PCR	Polymerase chain reaction
PFA	Paraformaldehyde
PI3-Akt	Phosphoinositide 3-kinase-protein kinase B
PSD95	Post- synaptic density protein 95
RT	Room temperature
RT-PCR	Reverse transcriptase-polymerase chain reaction
RTT	Rett Syndrome
SEM	Standard error of the mean
SNAP25/*Snap25*	Synaptosome- associated protein 25 (protein/mouse gene)
THAL	Thalamus
TrkB	Tropomyosin receptor kinase B
WB	Western blot
XCI	X-chromosome inactivation
