# Supplementary material for: Mutation of MeCP2 at T158M Leads to Distinct Molecular and Phenotypic Abnormalities in Male and Female Mice
Source: Cells. 2025 Aug 19;14(16):1286. doi: 10.3390/cells14161286 (PMC12384606; doi:10.3390/cells14161286)
Supplement: Supplementary file 1 [file cells-14-01286-s001.zip › cells-3645703-supplementary.pdf]

Supplementary Figures

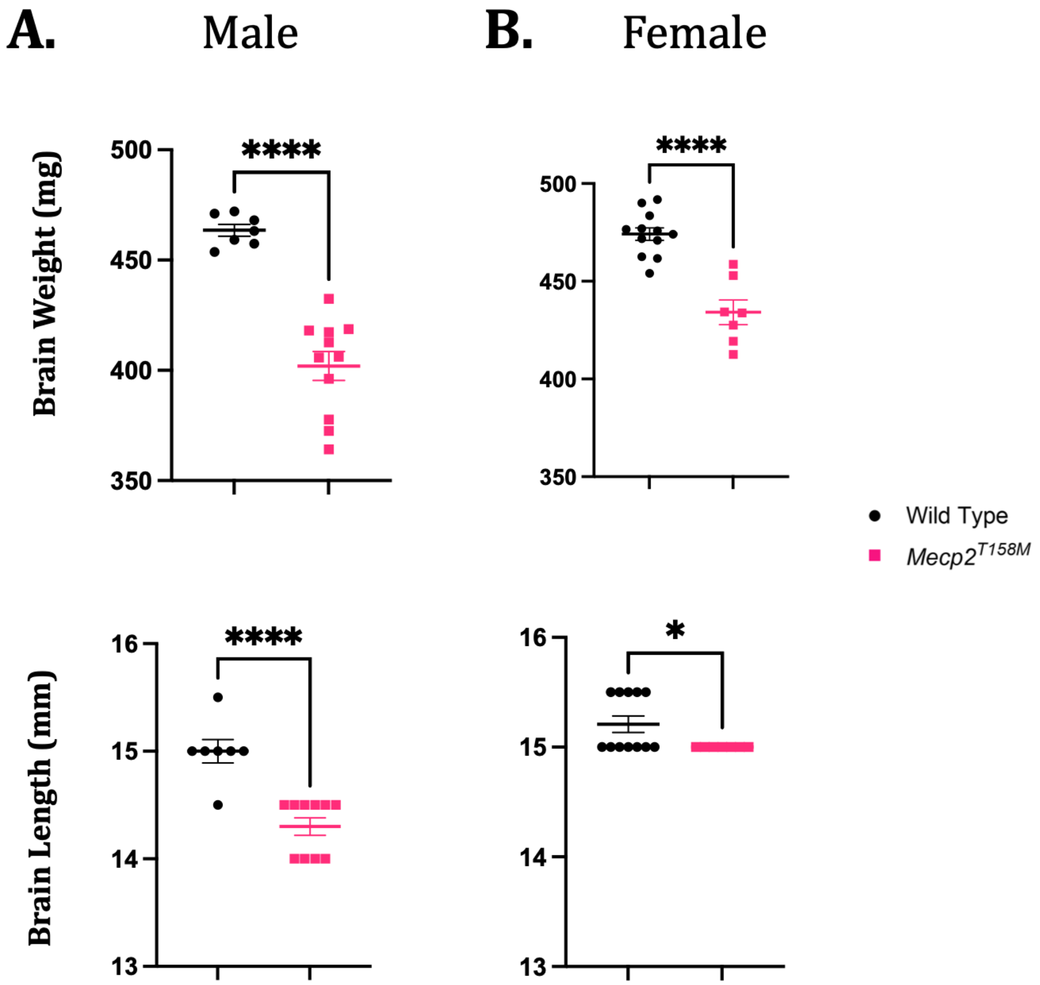

Figure S1. Comparative analysis of the mean brain weight (mg) and length (mm) of male and female *Mecp2*^{T158M} mice and their age- and sex-matched wild-type controls. Studies were completed at the time of RTT-like phenotype in the mutant mice (7-9 weeks males, 18-19 weeks females). (A-I) Analysis of the brain weight in the *Mecp2*^{T158M} male mice and controls. (A-II) Analysis of the brain length in the *Mecp2*^{T158M} male mice and controls. B-I and B-II. Similar analysis as (A-I) and (A-II) for female *Mecp2*^{T158M} and control mice. Statistical analysis was performed by unpaired *t*-test in each sex and presented as mean ± SEM. For mouse brain weight, experimental groups include male wild-type (n = 7) and hemizygous *Mecp2*^{T158M} (n = 11) mice; and female wild-type (n = 12) and heterozygous *Mecp2*^{T158M} (n = 7) mice. For mouse brain length, experimental groups include male wild-type (n = 7) and hemizygous *Mecp2*^{T158M} (n = 10) mice; and female wild-type (n = 12) and heterozygous *Mecp2*^{T158M} (n = 7) mice. Degrees of significance are shown as: $*p < 0.05$ and $****p < 0.0001$.

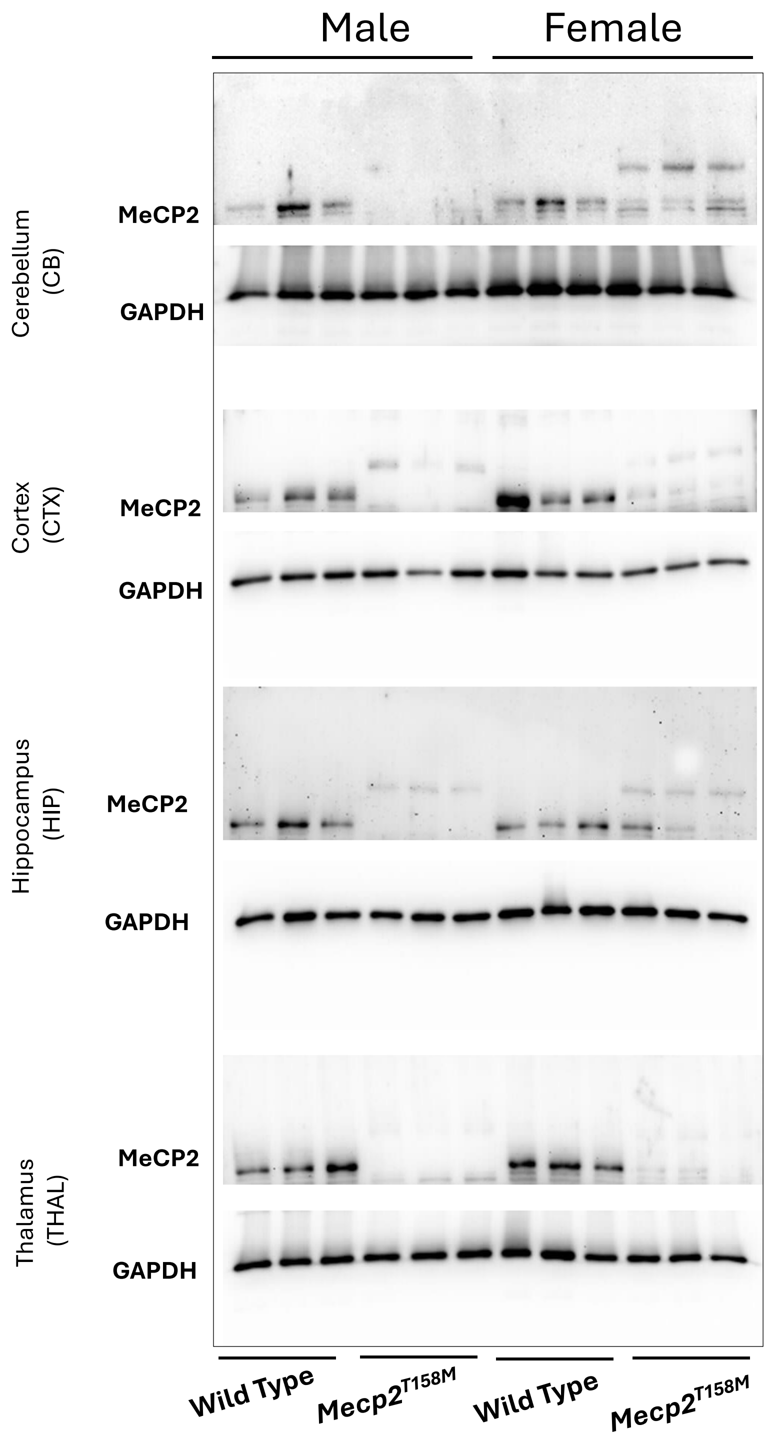

Figure S2. Uncropped images of Western blot signals for Figure 1. Note that all membranes were re-probed for GAPDH, and the GAPDH signals for each MeCP2 membrane in the cerebellum, cortex, hippocampus and thalamus are shown. Uncropped images of GAPDH signals are also shown here. All proteins were quantified using their respective GAPDH for the corresponding membranes. Signal quantifications are provided in Figure 1.

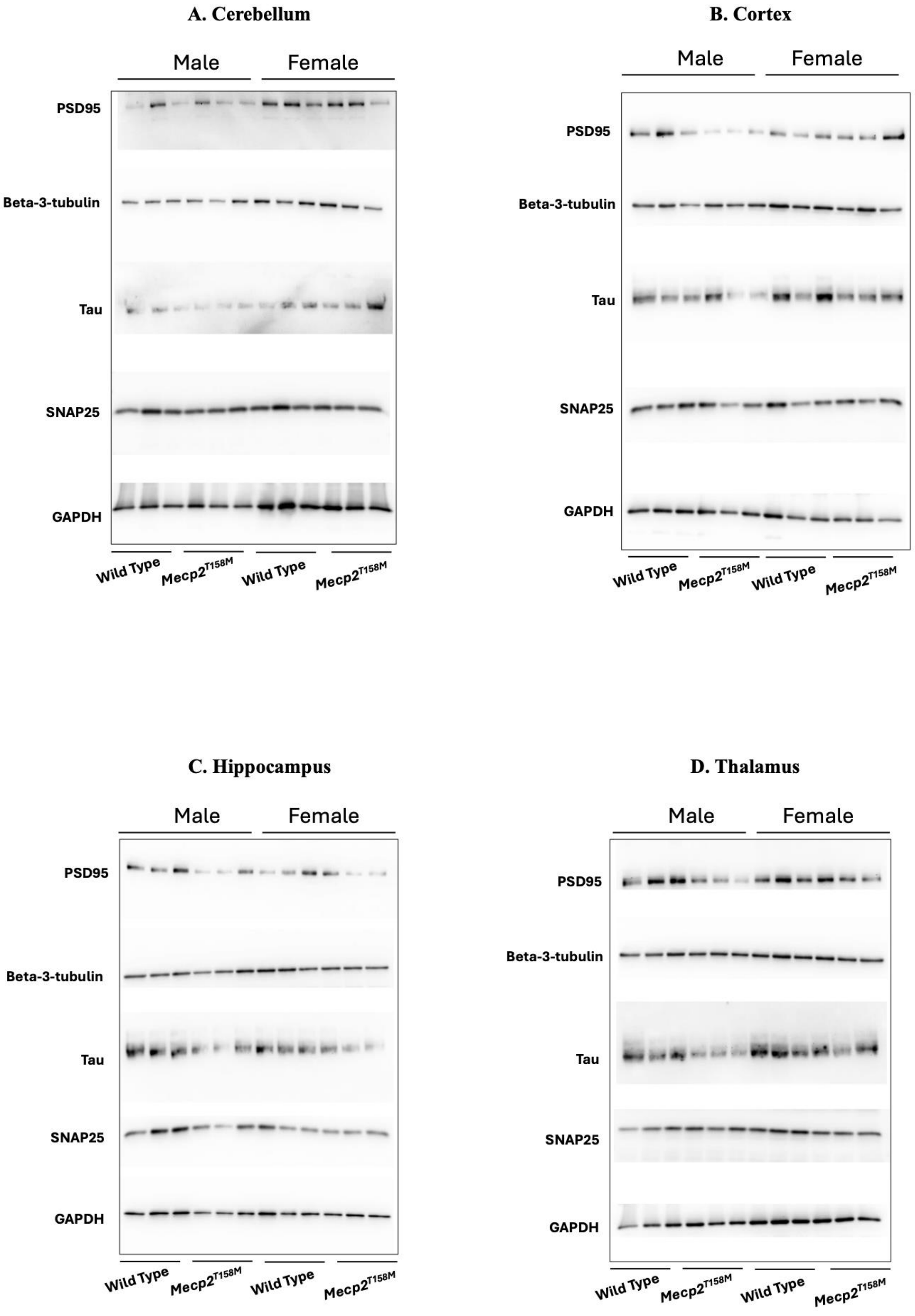

Figure S3. Uncropped images of Western blot signals for Figure 2. Note that all membranes were re-probed for GAPDH, but only one GAPDH representative is shown in Figure 2 for each of the four brain regions. Uncropped images of GAPDH signals are also shown here. All proteins were quantified using their respective GAPDH for the corresponding membranes. Signal quantifications are provided in Figure 2.

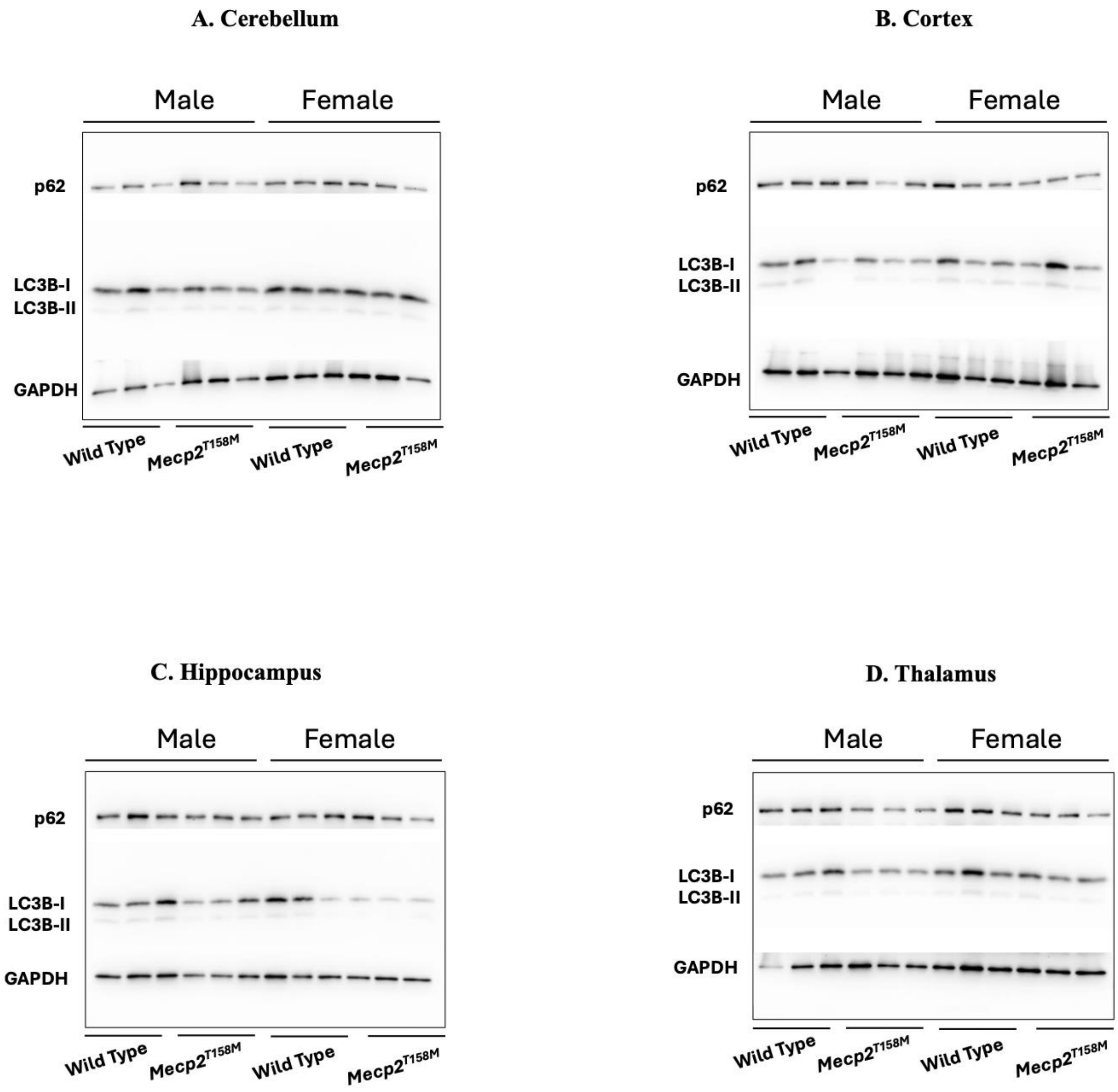

Figure S4. Uncropped images of Western blot signals for Figure 3. Note that all membranes were re-probed for GAPDH, but only one GAPDH representative is shown in Figure 3 for each of the four brain regions. Uncropped images of GAPDH signals are also shown here. All proteins were quantified using their respective GAPDH for the corresponding membranes. Signal quantifications are provided in Figure 3.

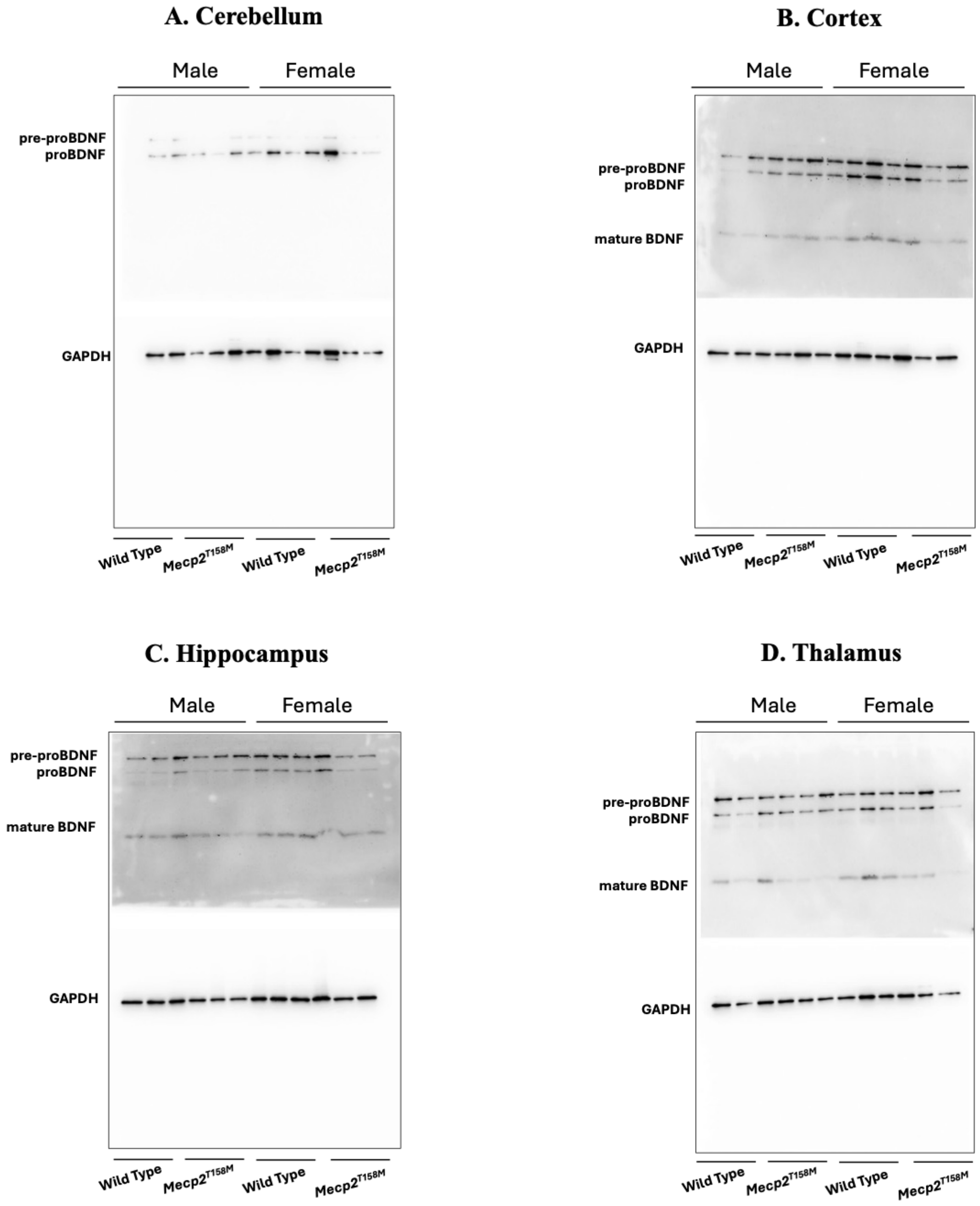

Figure S5. Uncropped images of Western blot signals for Figure 4. Note that all membranes were re-probed for GAPDH, and the GAPDH signals for each BDNF membrane in the cerebellum, cortex, hippocampus and thalamus are shown. Uncropped images of GAPDH signals are also shown here. All proteins were quantified using their respective GAPDH for the corresponding membranes. Signal quantifications are provided in Figure 4.

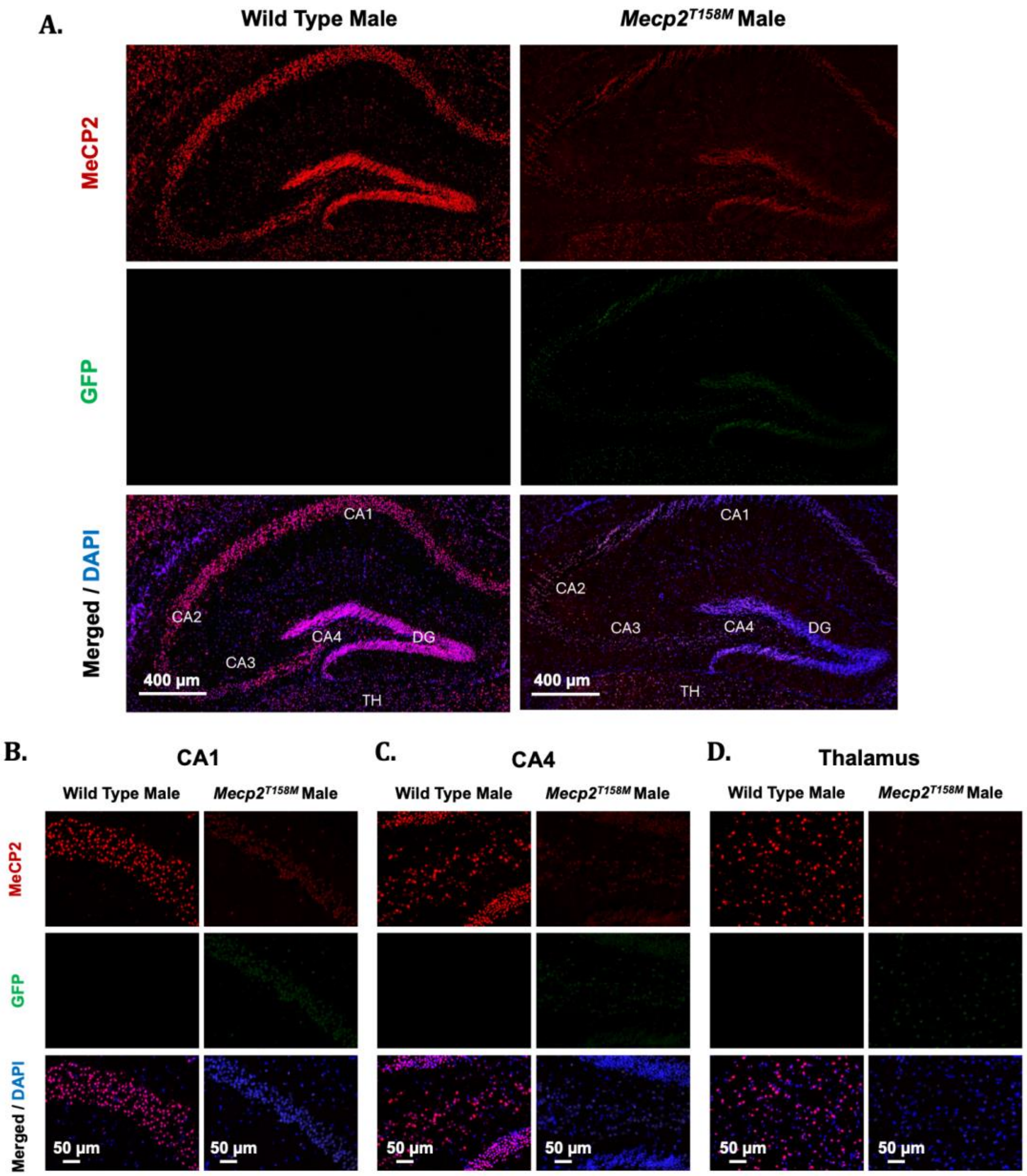

Figure S6. MeCP2 expression in the brain of male *Mecp2*^{T158M} mice and wild-type controls. Representative images are shown for immunohistochemical assessment of brain tissues in the hippocampus and thalamus, mice were used at approximately 8 weeks of age (A-D). (A) Signals are shown for MeCP2 using an antibody against MeCP2 C-terminal region. Signals are also shown for protein detection by an anti-GFP antibody in the hippocampus and thalamus of male wild-type and *Mecp2*^{T158M} mice. Panel (B) shows MeCP2 and GFP in the CA1 region of the hippocampus of male wild-type and *Mecp2*^{T158M} mice. Panel (C) shows MeCP2 and GFP in the CA4 region of the hippocampus of male wild-type and *Mecp2*^{T158M} mice. Panel (D) shows MeCP2 and GFP in the thalamus of male wild-type and *Mecp2*^{T158M} mice. Experiments were completed in three wild-type and two *Mecp2*^{T158M} mice. In (A), the layers of the hippocampus (DG: dentate gyrus, CA1: hippocampal cornu ammonis 1, CA2: hippocampal cornu ammonis 2, CA3: hippocampal cornu ammonis 3, and CA4: hilus) and TH: thalamus are indicated in white text. In (A-D), MeCP2 (red), GFP (green), and the overlay with DAPI (blue) are shown. Scale bars represent 400 µm in (A) and 50 µm in (B-D). Primary antibody omission images are shown in Supplementary Figure S8.

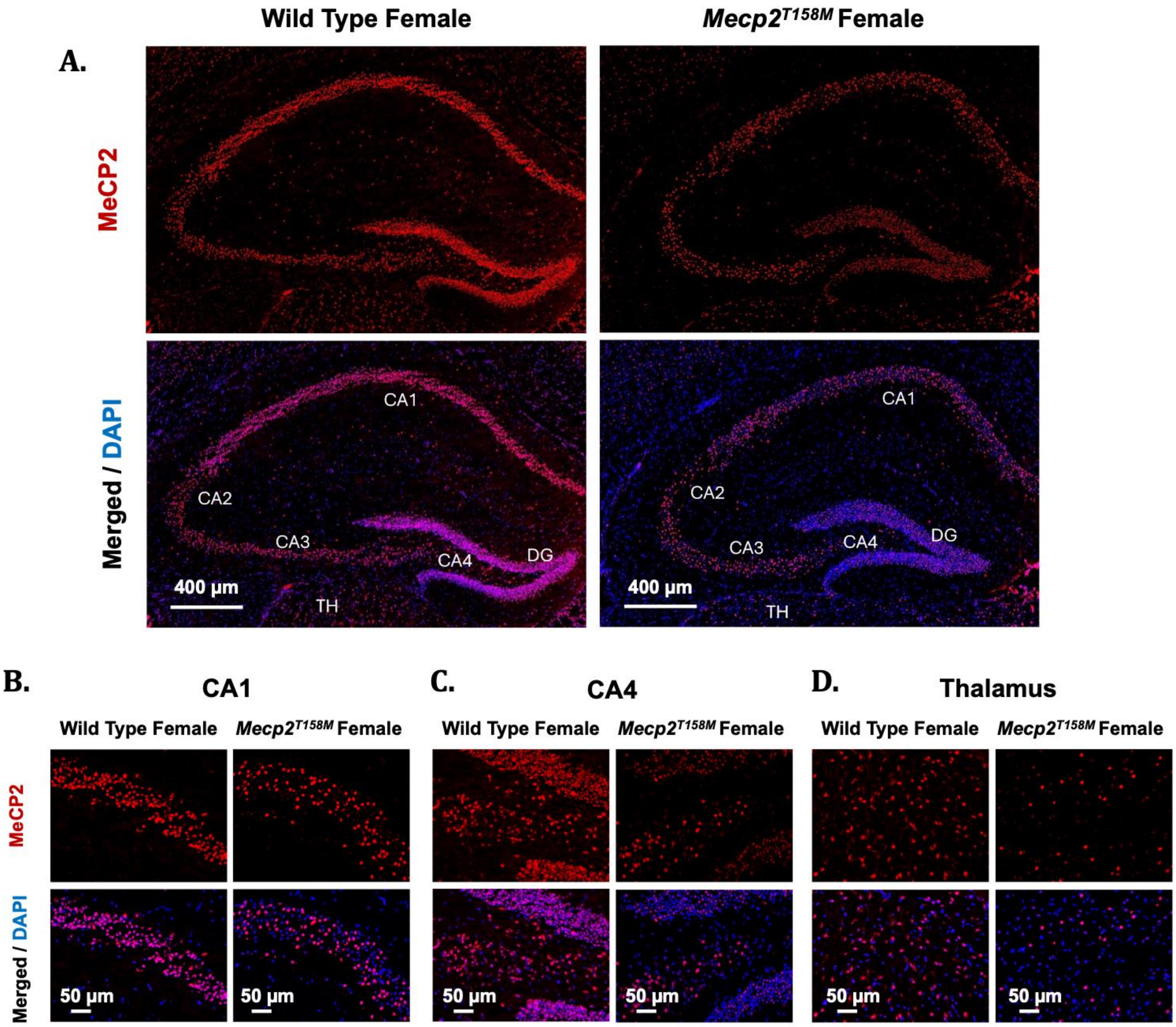

Figure S7. MeCP2 expression in the brain of female *Mecp2*^{T158M} mice and wild-type controls. Representative images are shown for immunohistochemical assessment of brain tissues in the hippocampus and thalamus, mice were used at approximately 17-18 weeks of age (A-D). (A) Signals are shown for MeCP2 using an antibody against MeCP2 N-terminal region. Panel (B) shows MeCP2 in the CA1 region of the hippocampus of female wild-type and *Mecp2*^{T158M} mice. Panel (C) shows MeCP2 in the CA4 region of the hippocampus of female wild-type and *Mecp2*^{T158M} mice. Panel (D) shows MeCP2 in the thalamus of female wild-type and *Mecp2*^{T158M} mice. Experiments were completed in two wild-type and three *Mecp2*^{T158M} mice. In (A), the layers of the hippocampus (DG: dentate gyrus, CA1: hippocampal cornu ammonis 1, CA2: hippocampal cornu ammonis 2, CA3: hippocampal cornu ammonis 3, and CA4: hilus) and TH: thalamus are indicated in white text. In (A-D), MeCP2 (red) and the overlay with DAPI (blue) are shown. Scale bar represents 400 μm in (A) and 50 μm in (B-D). Primary antibody omission images are shown in Supplementary Figure S9.

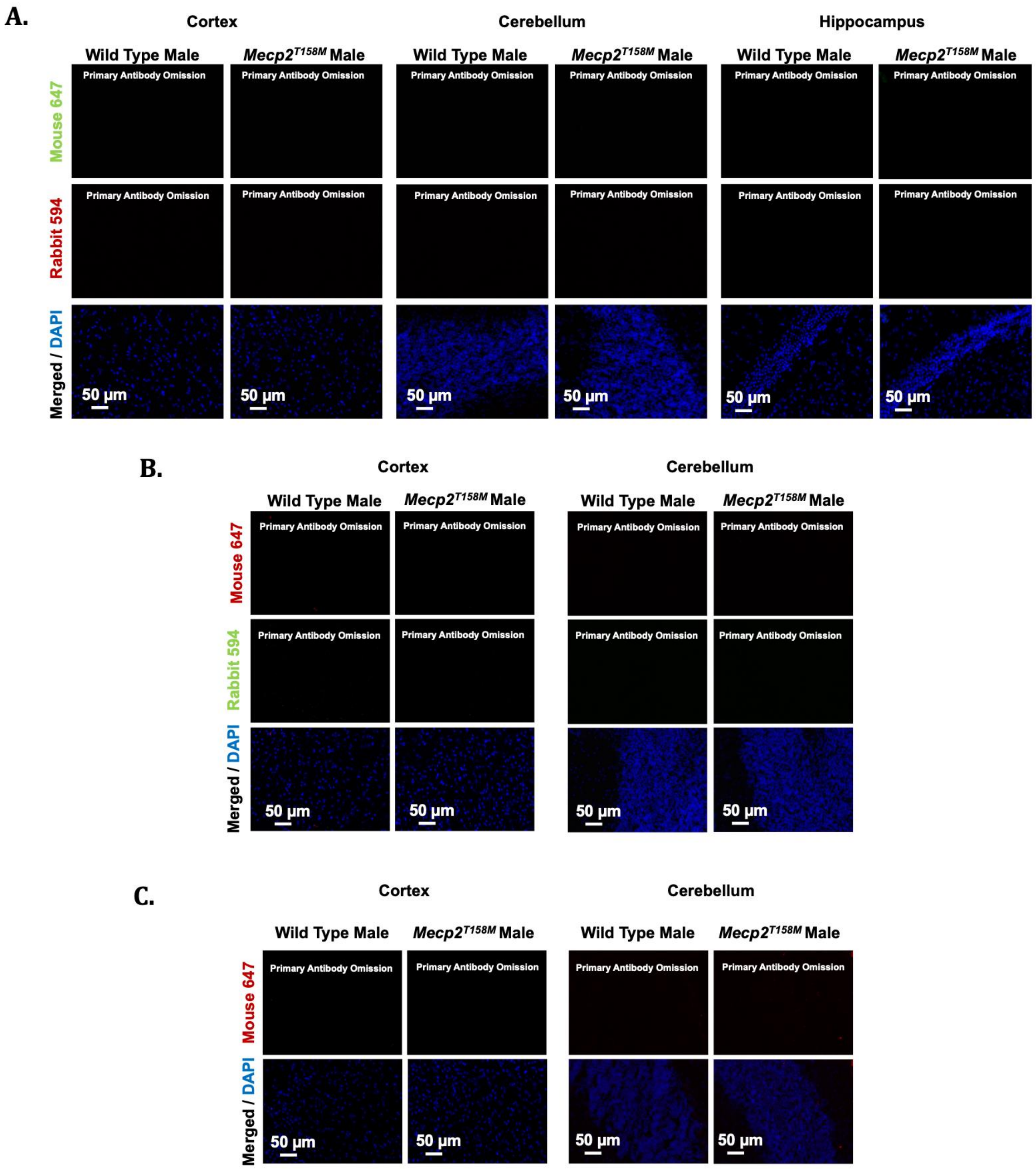

Figure S8. Controls verifying specificity of immunolabelling in the brain of male *Mecp2*^{T158M} mice and wild-type controls. Mice were used at approximately 8 weeks of age. (A) Primary omission controls and DAPI (blue) for MeCP2 (C-terminal region) and GFP in the cortex, cerebellum and hippocampus. (B) Primary omission controls and DAPI (blue) for SNAP25 and β-III-Tubulin in the cortex and cerebellum. (C) Primary omission controls and DAPI (blue) for MeCP2 (N-terminal region) in the cortex and cerebellum.

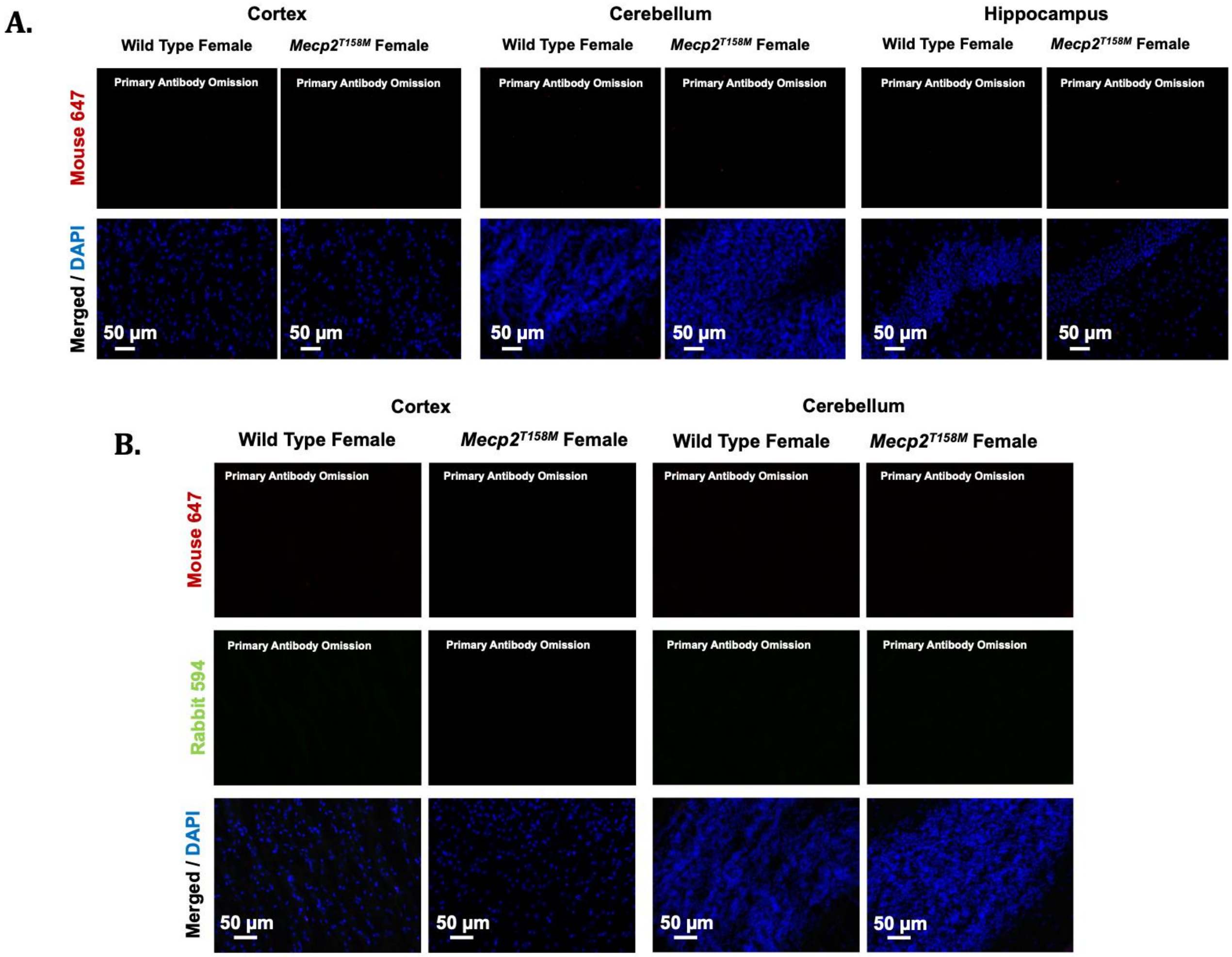

Figure S9. Controls verifying specificity of immunolabelling in the brain of female *Mecp2*^{T158M} mice and wild-type controls. Mice were used at approximately 17-18 weeks of age. (A) Primary omission controls for MeCP2 (N-terminal region) in the cortex, cerebellum, and hippocampus. (B) Primary omission controls for SNAP25 and β-III-Tubulin in the cortex and cerebellum.

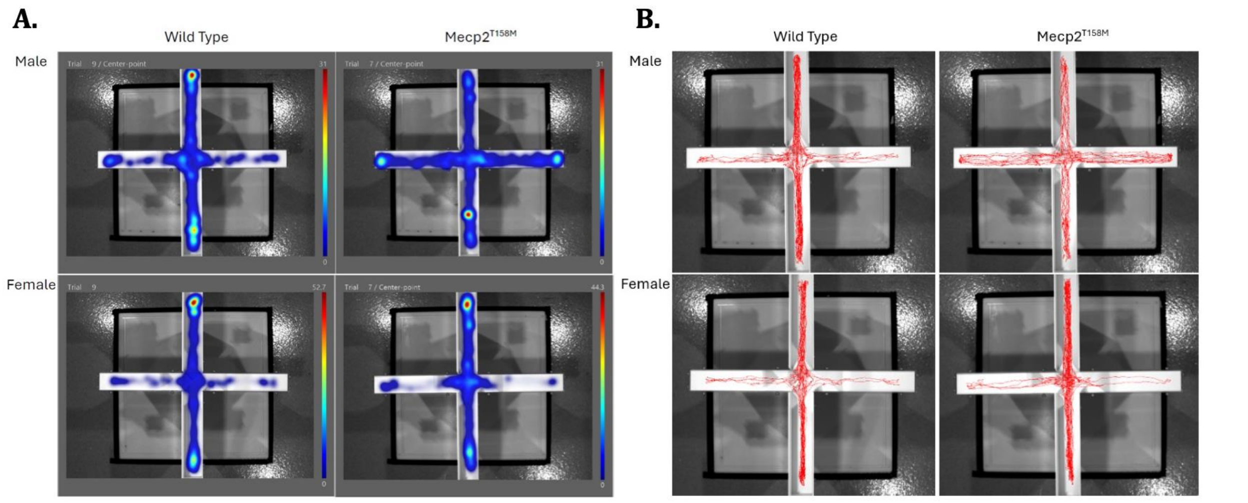

Figure S10. Heat and tracking maps for the elevated plus maze (EPM) for wild-type and *Mecp2*^{T158M} males and females. (A) Representative heat maps are shown for the time spent in the different areas of the elevated plus maze, where a blue color represents the least amount of time spent in the area while a warmer (red/orange) color represents a greater time spent in that area. (B) Representative track maps are shown for the paths taken by the mouse as well as their movement.

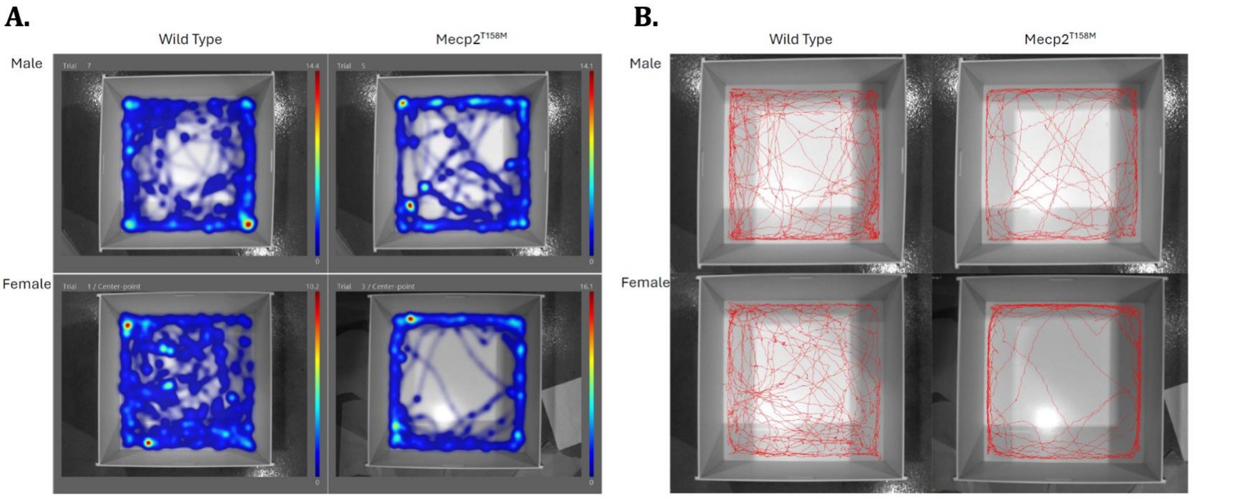

Figure S11. Heat and track visualization maps for the open field test (OFT) for wild-type and *Mecp2*^{T158M} males and females. (A) Representative heat maps are shown for the time spent in the different areas of the open field box, where a blue color represents the least amount of time spent in the area while a warmer (red/orange) color represents a greater time spent in that area. (B) Representative track maps are shown for the paths taken by the mouse as well as their movement.

Table S1. Scoring reference and endpoint monitoring checklist.

**Animal ID#**								
**Species/strain**			**Protocol**					
**Room**			**PI**					
**Experimental Treatment Group**								
**Date**								
**Weight (grams)**								
**% of peak weight**								
**Experimental Treatment given (ip.)**								
**1) Activity/mobility**								
**2) Gait**								
**3) Hind-limb clasping**								
**4) Tremor**								
**5) General condition (appearance)**								
**6) Breathing**								
Final breathing score (0, 1 or 2)								
**Tooth condition**								
**Food/Water consumption**								
**Treatment given? (remedial)**								
**Comments (injection time & side)**								
**Humane endpoint reached (Y/N)**								
**Initials**								

For scoring 1) through 6), write a 0, 1 or 2 based on observations.

Table S2. Antibodies used for Western blot and immunohistochemistry experiments.

Antibody	Dilution	Source
Western blotting primary and secondary antibodies		
anti-MeCP2 (rabbit polyclonal)	1:1000	Sigma-Aldrich, ABN1728
anti-GAPDH (rabbit polyclonal)	1:5000	Sigma-Aldrich, G9545
anti-SNAP25 (rabbit polyclonal)	1:1000	Sigma-Aldrich, S9684
anti-BDNF (rabbit monoclonal)	1:1000	Abcam, ab108319
anti-SQSTM1/p62 (rabbit polyclonal)	1:1000	Cell Signaling, 5114S
anti-LC3A/B (rabbit monoclonal)	1:1000	Cell Signaling, 12741P
anti-PSD95 (mouse monoclonal)	1:1000	Invitrogen, MA1045
Anti-Rabbit IgG (secondary HRP-linked)	1:5000	Cell Signaling, 7074P2
Anti-Mouse IgG (secondary HRP-linked)	1:5000	Cell Signaling, 7076P2
Immunohistochemistry primary and secondary antibodies		
MeCP2 (D4F3) XP® (C-terminal, rabbit monoclonal)	1:500	Cell Signaling, 3456
anti-MeCP2 (N-terminal, mouse monoclonal)	1:200	Sigma-Aldrich, M7443
anti-SNAP25 (rabbit polyclonal)	1:1500	Sigma-Aldrich, S9684
anti-p62/SQTM1 (rabbit polyclonal	1:500	Sigma-Aldrich, P0067
Anti-Green Fluorescent Protein (GFP) Antibody (mouse monoclonal)	1:400	Sigma-Aldrich, MAB3580
Anti-β-Tubulin III Antibody (Mouse monoclonal)	1:800	Sigma-Aldrich, T8578
Goat anti-Mouse IgG (H+L) Highly Cross-Adsorbed Secondary Antibody, Alexa Fluor™ 594	1:500	Invitrogen/Thermo Fisher Scientific, A11032
Goat anti-Mouse IgG (H+L) Highly Cross-Adsorbed Secondary Antibody, Alexa Fluor™ 647	1:500	Invitrogen/Thermo Fisher Scientific, A32728
Goat anti-Rabbit IgG (H+L) Highly Cross-Adsorbed Secondary Antibody, Alexa Fluor™ Plus 488	1:500	Invitrogen/Thermo Fisher Scientific, A32731
Goat anti-Rabbit IgG (H+L) Cross-Adsorbed Secondary Antibody, Alexa Fluor™ 594	1:500	Invitrogen/Thermo Fisher Scientific, A11012
Goat anti-Rabbit IgG (H+L) Cross-Adsorbed Secondary Antibody, Alexa Fluor™ 647	1:500	Invitrogen/Thermo Fisher Scientific, A21244
